# Impact of Phage Therapy on *Pseudomonas syringae* pv. *syringae* and Plant Microbiome Dynamics Through Coevolution and Field Experiments

**DOI:** 10.1111/1462-2920.70076

**Published:** 2025-03-12

**Authors:** Matevz Papp‐Rupar, Emily R. Grace, Naina Korotania, Maria‐Laura Ciusa, Robert W. Jackson, Mojgan Rabiey

**Affiliations:** ^1^ NIAB EMR West Malling UK; ^2^ School of Biosciences and the Birmingham Institute of Forest Research University of Birmingham Edgbaston UK; ^3^ School of Life Sciences University of Warwick, Gibbet Hill Campus Coventry UK; ^4^ School of Life Sciences University of Warwick, Innovation Campus Stratford‐upon‐Avon UK

**Keywords:** bacteriophage, biocontrol, cherry canker, microbiome, *Pseudomonas syringae*

## Abstract

Bacteriophages (phages) are viruses that infect and lyse bacteria and have the potential for controlling bacterial diseases. Isolation of phages targeting the cherry pathogen *Pseudomonas syringae* pv. *syringae* (*Pss*) led to five distinct phage genotypes. Building on previous in vitro coevolution experiments, the coevolution of the five phages (individually and as a cocktail) with *Pss* on cherry leaves was conducted in glasshouse and field experiments. Phages effectively reduced *Pss* numbers on detached leaves, with no evidence of phage resistance emerging in the bacterial population. Field application of phages in a cherry orchard in Southeast England evaluated phage survival, viability and impact on bacterial populations and the microbial community. The bacterial population and phages persisted in the leaf and shoot environment as long as the bacterial host was present. In contrast to in vitro studies, the plant environment constrained the emergence of phage resistant *Pss* populations. Application of phage cocktail in the orchard did not affect the cherry leaf microbiome. These observations provide essential knowledge for using phage treatments to control bacterial diseases while minimising the impact on the plant microbiome, highlighting phages' potential to safely control bacterial diseases in trees.

## Graphical abstract

The coevolution of phages with *Pseudomonas syringae* on cherry leaves reduced bacterial numbers on detached leaves without resistance emerging. Field application of phages in a cherry orchard showed that phages and bacteria persisted as long as the bacterial host was present, with the plant environment limiting the emergence of phage resistant populations.

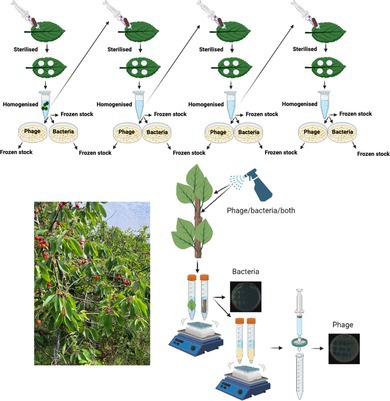

## 1 Introduction

*Pseudomonas syringae* is a member of a species complex of bacterial plant pathogens infecting over 180 plant species, including crucial crops, and posing a significant economic threat (Xin et al. 2018). *Prunus* species, such as cherry ( *Prunus avium* ), are highly susceptible to *P. syringae* , leading to bacterial canker that can severely damage and kill the tree and thus significantly impact commercial cherry and stone fruit production. Three causative agents of cherry canker have been identified: *P. syringae* pathovars *syringae* (*Pss*) and *morsprunorum* race 1, and race 2 (*Psm*1 and *Psm*2) (Bultreys and Kaluzna 2010). Managing bacterial canker in *Prunus* presents challenges due to variations in tolerance and susceptibility between cultivars and the evolution of virulent strains of the pathogen (Jones et al. 2007). Traditional antimicrobial controls based on streptomycin and copper‐based treatments are ineffective and drive pathogen resistance to these products, with rapid spread of genetic elements in pathogen communities. This has led to the curtailment of use via regulation to reduce the use and spread of resistance genes (Stone and Baker 2010; Sundin and Wang 2018). Cherry tree susceptibility and tolerance to pathogens are complex, thus breeding for resistance is difficult, requiring screening for multiple pathogens and accounting for their varied aggressiveness towards cherry's defence mechanisms (Farhadfar et al. 2016; Hulin et al. 2018).

Given these challenges, innovative methods of disease control must be considered, including bacteriophage biocontrol. Bacteriophages (phages) are viruses that target bacteria, causing lysis without harming plants or animals. Phages show specificity to bacterial hosts and have demonstrated potential as biocontrol agents against *P. syringae* pathovars (James et al. 2020; Rombouts et al. 2016; Warring et al. 2022). Their specificity, lytic cycle and abundance in the environment position phages as promising candidates for treating bacterial infections in plants and animals.

In a previous study, 13 phages known as MR phages were characterised that efficiently reduced *Pss*, *Psm*1, and *Psm*2 populations (Rabiey et al. 2020). Genome sequencing identified five genomically distinct phage genotypes among these phages. While in vitro analysis revealed significant reductions in *Pss* populations caused by phage, bacterial resistance emerged using rich nutrient media. Further in vitro experiments exploring the coevolution of phages and bacteria in vitro with five MR phages individually and in combination of five (cocktail 5C) showed *Pss* developing mechanisms to resist phage infection. However, these experiments were conducted in highly controlled experimental conditions (constant temperature, rich nutrient medium, shaking environment) not representative of a varying plant tissue environment that has changes in temperature, moisture, UV light and host resistance mechanisms, as well as competition pressure from other native microbes. This raises questions about whether phage resistance emerges in the natural plant environment, which can be tested under semi‐controlled (*in planta*) and natural field conditions.

One of the few studies exploring phage resistance *in planta* is Hernandez and Koskella (2019), using *Pseudomonas syringae* pathovar *tomato* (*Pst*), lytic phages FRS and SHL and tomato plants. Despite extensive screening and long‐term exposure of phages to *Pst* within leaf tissues, phage resistant *Pst* isolates were only rarely detected, if at all. However, there is evidence of phage resistance occurring against human bacterial pathogens in vivo (Castledine et al. 2022; Liu, Tian, et al. 2022). Several studies have instead explored phage resistance mutants which have emerged in vitro, either via spontaneous mutation or transposon libraries, and compared their fitness traits to the ancestral (i.e., wild type) bacteria. The impact of phage resistance in these studies can vary dependent on the fitness trait measured, the bacterial species used, and the mutation that confers resistance (Bartnik et al. 2022; Hernandez and Koskella 2019; Wang et al. 2023; Zhang et al. 2022). Importantly, however, some studies have identified a significant reduction in the virulence of phage resistant mutants compared to the ancestral bacteria, for example in phage resistant *Xanthomonas oryzae* pv. *oryzae* on rice leaves, *Dickeya solani* on potato tubers, and *Ralstonia solanacearum* on tomato (Bartnik et al. 2022; Wang et al. 2023; Zhang et al. 2022). Based on these observations, our overarching hypothesis was that the application of *Pss*‐specific phages *in planta* would not result in the emergence of phage resistance, significant changes to *Pss* fitness, or significant alterations to plant microbiomes.

This study evaluated phage‐*Pss* interactions *in planta* on cherry leaves and shoots to investigate the outcomes of phage therapy in agroecosystems. We sought to understand whether the application of phage cocktail 5C to *Pss* inoculated leaves and shoots led to the emergence of bacterial phage resistance and changes in pathogen fitness (defined as bacterial growth relative to wildtype). We also measured the fate of the phage applied to the plant tissue to determine if it was stable and viable within a cherry orchard. Finally, we examined the plant leaf microbiome before and after phage treatment to determine whether phage application impacted bacterial and fungal populations and microbiome density, diversity and composition. Understanding phage‐pathogen‐microbiome dynamics is crucial for developing phage biocontrol as a safe and precise treatment approach to control disease in agroecosystems.

## 2 Material and Methods

### 2.1 Bacteria and Phage Culture

*Pss* strain 9097 grown to an optical density (OD_600_) of 0.2 at 600 nm (≃2 × 10^8^ bacterial cells per mL) was used in this study. Kings Medium B (KB or with added agar [KBA], King, Ward, and Raney 1954) was used to culture *Pss*. Five *Pss‐*infecting phages, collected and characterised by Rabiey et al. (2020), were used in this study: MR1, MR4, MR6, MR14, MR15 and cocktail of all five phages (cocktail 5C). These phages were selected as representatives of the five distinct genomotypes identified in the detailed analysis of phage genomes by Rabiey et al. (2020). Phages were stored and diluted using phosphate‐buffered saline (PBS, Sigma). KB with 0.7% agar was used in the soft top agar overlay for the phage assays.

Phages were amplified by plating 10^6^ plaque forming units (PFU) per mL (to have a clear lawn) stocks with *Pss* on a soft agar overlay plate. After overnight incubation at 27°C, 5 mL of PBS was added onto the plate and incubated at room temperature for 1 h with agitation every 15 min. The liquid was removed and filtered through a 0.22 μm filter to remove any bacteria. Phage titre was determined via a spot assay at different dilutions before storage at 4°C.

### 2.2 Coevolution of *Pss* and Phages on Detached Cherry Leaves

Coevolution of *Pss* and each MR phage individually and cocktail 5C was performed on detached freshly picked (2‐ to 3‐week‐old) cherry leaves. The detached leaves were surface sterilised with 70% ethanol and allowed to air dry, and infiltrated with a bacterial suspension of *Pss* (2 × 10^8^ CFU mL^−1^), either single or a cocktail of phages (10^8^ PFU mL^−1^), or PBS (negative control) on the abaxial surface using a blunt‐ended 1 mL syringe. Leaves were placed in transparent boxes with lids in a plant growth chamber and incubated for 72 h at 22°C (16:8 h, light:dark). Each leaf was infiltrated four times using the same treatment, and at least three leaves were inoculated per treatment. Four discs (1 cm in diameter) were excised from each leaf and placed into a 2 mL tube containing 1 mL of PBS with two tungsten beads (Qiagen, UK) and homogenised at a speed of 4 m s^−1^ for 15 s. The tubes were centrifuged briefly at 4000 rpm to remove plant debris, and a serial dilution of the supernatant was prepared, with 10 μL spotted on KBA to measure CFU mL^−1^ of the bacterial population. For phage populations, the supernatant was filtered through a 0.22 μm filter and spotted on soft agar containing *Pss*. A 400 μL of each population was frozen at −80°C for further study. The homogenised supernatant was used to infiltrate a fresh set of detached leaves for the next passage, with passaging done four times in total. Both CFU and PFU were counted at each passage using a spot assay of 5 μL of either homogenate on KBA or filtered homogenate on a soft agar overlay. Each dilution was tested in triplicate (technical replicates).

### 2.3 Field Experiment

A field trial with phages was conducted between 19 June 2023 and 19 July 2023 and then 3 months after in September, at a cherry orchard research plot in NIAB, East Malling (N 51°  17′ 31.7″, E 0°  26′  52.1″). The trees used were 15‐year old *Prunus avium* cultivar Sweetheart on Gisela 5 rootstock. The distance between trees was 1.5 m, with a 3 m distance between each row. There were two untreated guard trees between every experimental tree.

A fully randomised block design with two factors (each with two levels), over five blocks with four plots per block (one tree per plot) was used. The two factors were ‘phages’ (cocktail 5C yes/no) and ‘*Pss*’ (*Pss* bacteria yes/no) for four total treatment groups: ‘Control’ consisting of PBS buffer; ‘cocktail 5C’ (10^8^ PFU mL^−1^); ‘*Pss*’ (2 × 10^8^ CFU mL^−1^); and a combination of ‘*Pss* + cocktail 5C’, where *Pss* was applied first followed by cocktail 5C within 12 h. On each tree, 12 healthy branches were selected at random. On six out of the twelve branches, treatments were applied to 4 cm of shoot and also to four randomly selected healthy leaves (both upper and lower surfaces); on the remaining six branches only 4 cm of shoot was sprayed with treatments. A hand‐held atomiser (sprayer) was used to apply three mists to each side of the leaf or shoot, approximately 50 μL per side. To prevent cross‐contamination, the order of treatments applied was as follows: PBS; *Pss*; cocktail 5C; *Pss* + cocktail 5C.

One day before treatment application, two random leaves and one random shoot (0.5–0.8 cm thick and 4 cm long) were collected from each experimental tree to assess the pre‐existing (baseline) levels of *Pss* and phage populations.

*Pss* was sprayed on leaves and shoots before 9 am on the treatment day using a hand‐held atomiser, with subsequent leaf sampling conducted 4 h after *Pss* application to confirm viability and uniformity of the bacterial inoculum. Cocktail 5C was sprayed after 7 PM of the same day to reduce the risk of phage degradation by UV irradiation and enable bacterial establishment. Subsequent sampling was done in the morning of day 1‐, 2‐, 3‐, 4‐ and 30‐day post treatment (DPT) to quantify *Pss* and phage populations. Three (out of four treated) random leaves from two shoots per tree were collected and combined into a single sample. Similarly, two random shoot sections (0.5–0.8 cm thick and 4 cm long) were collected from each tree at each time point and processed as described below to assess both *Pss* and phage population sizes and collect representative strains.

The leaves were cut into small pieces and placed in 10 mL PBS in 15 mL sterile tubes. Shoot sections were placed directly in 10 mL PBS in 15 mL sterile tubes. After shaking at 27°C at 200 rpm for 1 h, serial dilutions of the leaf and shoot washes were prepared in PBS and plated (5 μL droplet in triplicates) on KBA plates containing 40 μg mL^−1^ cephalexin (Melford, UK) and 100 μg mL^−1^ cycloheximide (Melford, UK) to assess the number of *Pseudomonas* CFU in each sample. Following this, 5 mL of each leaf or shoot wash was transferred to tubes containing 5 mL of sterile KB media to amplify phages. After 5 h incubation at 27°C with 200 rpm shaking, the phage suspensions were filtered through a 0.22 μm filter (FisherScientific, UK). To quantify phage PFU, filtered suspensions were serially diluted and 5 μL plated on KBA soft agar overlay, in triplicate, containing *Pss* 9097. Frozen stocks of leaf and shoot washes in PBS (bacterial stock) and filtered phage suspensions in KB (phage stock) were prepared in 20% sterile glycerol and stored at −80°C.

### 2.4 Growth Curve Assay

At the end of each *in planta* coevolution passage and the field experiment, each bacterial stock was streaked onto a KBA plate. Three colonies were picked randomly per sample and re‐streaked twice to remove any phages. Frozen glycerol stocks were made from overnight cultures of each colony. Each colony was then tested via colony PCR to ensure that they were *Pss* isolates, and not a pre‐existing leaf coloniser. *Pss‐*specific primers were designed to target a *Pss* rhamnosyltransferase (accession NZ_CP026568.1, gene BK06_011245), amplifying a 755 bp product: forward primer sequence (5′ to 3′) GGTGGTGGATCCCAGCTTCGATACGCTGATCG and reverse primer sequence (5′ to 3′) GGTGGTTCTAGAGCTCAGCTTGTAGGCCAGC. A 100 μL of liquid culture of each culture was centrifuged for 1 min at 14,000 rpm, then resuspended in 100 μL of nuclease‐free water. A 1 μL of each sample was then placed in a 25 μL volume reaction, containing 1.25 μL of each primer, 12.5 μL of GoTaq Green Master Mix, and 9 μL of nuclease‐free water. The positive control was *Pss* 9097 DNA, and the negative control was nuclease‐free water. Samples were run under the following PCR conditions: initial denaturation at 95°C for 5 min; 35 cycles of denaturation at 95°C for 30 s, annealing at 55°C for 30 s, and extension at 72°C for 1 min; and a final extension step at 72°C for 5 min.

Once confirmed to be *Pss*, the growth of colonies isolated from leaves and shoots was compared with ancestral *Pss* 9097 in an in vitro assay to assess whether phage treatment caused changes in bacterial growth fitness from the *in planta* coevolution and field experiments. Overnight cultures were produced of individual bacterial colonies from the coevolutionary passaging and from the field experiment. Cultures underwent centrifugation at 5,000 rpm for 5 min, followed by the removal of the supernatant. The resulting pellet was resuspended in 1 mL of PBS and adjusted to OD600 of 0.2 before aliquoting 100 μL onto a Greiner 96‐well flat‐bottomed plate containing 100 μL KB. A 100 μL of PBS and 100 μL of KB served as the negative control. Optical density measurements at 600 nm for each bacterial colony were taken using a TECAN SPARK Multimode Microplate Reader at 20‐min intervals over a 24‐h period. The assay was conducted at 27°C with shaking for 10 s before each reading. The experiment incorporated three replicates.

### 2.5 Killing Curve Assay

To assess the ability of wildtype phages in lysing *Pss* colonies collected at different time points during the *in planta* coevolution and field experiments, killing curve assays were conducted using the TECAN SPARK Multimode Microplate Reader. Three colonies from each replicate for both experiments were examined for their susceptibility to the original phage treatment they encountered. For instance, colonies from cocktail 5C‐treated *Pss* populations were tested against wildtype phage cocktail 5C. In each well of a Greiner 96‐well flat‐bottomed plate, 100 μL of *Pss* and 100 μL of individual phage combinations, at a multiplicity of infection (MOI) of 0.01, were dispensed. Positive controls consisted of 100 μL of *Pss* and 100 μL of KB, while negative controls contained 100 μL of PBS and 100 μL of KB. Measurements were taken at 600 nm at 20‐min intervals over 24‐h, with shaking for 10 s before each reading. The experiment incorporated three replicates.

### 2.6 *In Planta* Bacterial Growth Assay of *Pss* Isolates on Detached Cherry Leaves

Following the *in planta* coevolution experiments, detached leaf inoculation was performed following Hulin et al. (2023). Leaves were infiltrated as described above. In brief, freshly picked leaves (1‐ to 2‐week‐old) were infiltrated with bacterial suspension (2 × 10^6^ CFU mL^−1^) or with 10 mM MgCl_2_ as control, to the abaxial surface using a blunt‐ended 1 mL syringe. Leaves were then incubated as described previously before assessment. The leaves were then incubated for 7 days. After incubation, infiltrated leaf discs (four discs) were homogenised in 1 mL of 10 mM MgCl_2_ solution. To determine bacterial concentration (CFU mL^−1^), a dilution series was prepared and plated to allow single colony counting. Each dilution was tested in triplicate (technical replicates). Each leaf was infiltrated four times using the same strain, and at least three leaves were inoculated with each strain.

### 2.7 The Effect of Phages on Bacterial (16S) and Fungal (ITS) Microbiome in the Field

Using samples obtained from the field experiment, DNA extraction was performed on the remaining 5 mL of leaf washes, from all samples at 3 DPT and 30 DPT, for a total of 40 samples. Three DPT was chosen as the changes in bacterial CFU and phage PFU were detectable from this time point onward. Leaf washes were centrifuged at 5000 *g* for 20 min at 4°C, resuspended in 500 μL of sterile PBS, and stored in sterile 2 mL tubes at −20°C. All samples were centrifuged (16,000 g, 10 min) and the supernatant was carefully discarded. Each pellet (ca. 5 mg) was resuspended in 400 μL of lysis buffer, and the DNA was extracted according to the DNeasy Plant Mini Kit (Qiagen, Hilden, Germany) manufacturer's protocol, including the optional RNase A digestion step after lysis. Yields were analysed using a Nanodrop spectrophotometer (Thermo Scientific, Waltham, MA, US). The total bacterial (16S) and fungal (ITS) microbiome abundance was quantified using quantitative PCR (qPCR) using the same primer pairs as used in amplicon sequencing below (Papp‐Rupar et al. 2022). The effect of phages on the total 16S and ITS microbiome sizes (as log10 copy number) was analysed using the same statistical approach as in the analysis of CFU and PFU data.

The samples were sent to Novogene UK (Cambridge, UK) for PCR, library prep and amplicon sequencing of fungal ITS1 amplicon using ITS1‐1F (5′‐CTTGGTCATTTAGAGGAAGTAA‐3′, Gardes and Bruns 1993) and ITS2 primer (5′‐GCTGCGTTCTTCATCGATGC‐3′, White et al. 1990); and bacterial 16S V5‐V7 amplicon using 799F (5′‐AACMGGATTAGATACCCKG‐3′, Chelius and Triplett 2001) and 1193R (5′‐ACGTCATCCCCACCTTCC‐3′, Bodenhausen et al. 2013). Samples were sequenced on an Illumina NovaSeq platform in paired end mode with read length of 250 nt.

The bioinformatics and computational analyses were performed on CropDiversity‐HPC, described by Percival‐Alwyn et al. (2024). Amplicon sequence variants (ASVs) were generated from a combined set of leaf and shoot samples (across both 3 DPT and 30 DPT) but analysed separately, using a previously published pipeline (Papp‐Rupar et al. 2022). The following raw sequence reads were discarded in quality control step: reads with incorrect bases in the barcode or primer regions; and reads containing adapter contamination. Forward and reverse reads used in the ASV generation step were merged using the UPARSE pipeline V. 11.0 (Edgar 2013) with stringent criteria: minimum read length of 250 nt, zero differences in read overlap region, maximum expected error threshold of 0.2 (16S) and 0.1 (ITS) per sequence (Edgar and Flyvbjerg 2015) and minimal merged read length of 400 (16S) or 185 (ITS). Reads were then dereplicated, chimeric sequences removed and sequences with less than eight replicates discarded before generation of denoised ASVs. For frequency table generation, reads were merged using ‘differences in read overlap region’ set to 100 to ensure effectively all reads were merged. These unfiltered merged reads were aligned to the ASV representative sequences at the level of 97% similarity to produce an ASV frequency table. Finally, the SINTAX algorithm (https://www.drive5.com/usearch/manual/sintax_algo.html) was used to assign taxonomic ranks to each ASV with the Unite V8.3 (2021‐05‐10) fungal database (Kõljalg et al. 2013) and ‘the RDP training set V18’ database for the 16S rRNA gene (Cole et al. 2014). The SINTAX algorithm only resolves bacterial ASVs to the genus level, but may resolve fungal ASVs to the species level. Taxonomy assignment confidence was at the 80% level.

### 2.8 Statistical Analysis

#### 2.8.1 Bacterial (CFU) and Phage (PFU) Quantification Analysis

The bacterial CFU counts and phage PFU counts in 5 μL droplets were first averaged across three pseudo replicates per sample and log transformed (log10 + 1). The effect of treatment with cocktail 5C and *Pss*, and their interaction on the CFU and PFU counts was analysed using a mixed linear model using ‘lme4’ package (Bates et al. 2015) in RStudio (2023.12.0+369).

Leaf and shoot data were analysed separately using ‘Plot’ (unique plot ID, df = 20) as a random intercept to account for repeated measurements of the same plots at different time points. ‘Block’ (df = 4) and all interactions of ‘Time’ (DPT, df = 5) with ‘Phages’ (df = 1) and *Pss* (df = 1) were used as fixed factors. Package ‘emmeans’ (Lenth R 2024) was used to (a) estimate marginal means (emmeans) and standard error of the mean (SEM) for CFU and PFU counts in leaf and shoot, and (b) to compare each treatment group (df = 3) with every other treatment group within the same tissue and time point. The raw data and emmeans +/− SEM were plotted using ‘ggplot2’ (Wickham 2016) and ‘ggpubr’ (Kassambara 2023) packages.

#### 2.8.2 Growth, Killing Curve Analysis and *in Planta* Growth Analysis

To evaluate differences in the growth curves, killing curves and *in planta* growth analysis of the bacterial isolates in the absence and presence of phages, individually or in cocktail 5C, an ANOVA test was used. For the growth curves and killing curves, the analysis was done at specific time points, that is, at 500, 1000 and 1500 min. A post hoc Tukey test was applied to evaluate differences among treatments (*p* < 0.05). All the statistical analyses were carried out in GraphPad prism 9 (Boston, Massachusetts USA, www.graphpad.com), shown in Table S1.

#### 2.8.3 Microbiome Analysis

Amplicon analyses were carried out in RStudio (2023.12.0+369). Only the ASVs that cumulatively accounted for 99.5% of the total sequence reads were used in analysis. Before statistical analysis, the ASV count data were normalised for library size using ‘DESeq2’ package (Love et al. 2014) with the median‐of‐ratios (MR) method. The same fixed effects were used in all analyses, that is, ‘Block’ (df = 4) and all interactions of ‘Time’ (DPT, df = 5) with ‘5C’ (df = 1) and *Pss* (df = 1).

Chao1 (total estimated number of species per sample), Shannon's index (measure of microbiome diversity) and Simpson's index (measure of microbiome evenness) alpha diversity indices were calculated with the R ‘vegan’ 2.3‐1 package (Dixon 2003) and subjected to mixed‐effects permutation model analysis with ‘perm.lmer’ function in ‘permute’ package (Voeten 2023) to assess the main effects of Block, Time, 5C, *Pss* and two−/three‐way interactions of Time, 5C, *Pss*. Alpha diversity was assessed as the Chao1 measure (estimated total number of species per sample), Shannon's and Simpson's alpha diversity index. Shannon's index stresses the diversity and is more affected by rare taxa, whilst the Simpson index emphasises evenness and is more affected by the dominant taxa (Haines‐Young and Chopping 1996; Riitters et al. 2000).

Bray‐Curtis beta diversity indices were subjected to permutational multivariate ANOVA (PERMANOVA) to assess the effects of treatment factors (implemented as the Adonis function in the ‘vegan’ package). We used the ‘strata = Plot’ parameter to account for repeated measures of the same plots at 3 and 30 DPT.

ANOVA was applied to assess the treatment effects on the first five principal components (PCs) of both the bacterial and fungal microbiomes. Microbiome co‐occurrence networks of ASVs represented in PCs that were significantly affected by cocktail 5C were investigated further. First, we selected DEseq2 normalised counts of 50 ASVs with the highest absolute loading in PC. The data was split into subset of samples that received cocktail 5C (cocktail 5C, *Pss* + cocktail 5C) and those which did not (control, *Pss*). Correlation matrix (‘cor’ function in R) using Pearson's correlation coefficient and significance of each correlation (‘cor.test’ in R) was calculated separately for each subset of samples. Correlations with significance below 0.005 and absolute correlation coefficient above 0.3 were visualised using ‘igraph’ package in R.

‘Block’ factor was accounted for, and a Wald test was used in DEseq2 to identify specific ASVs with significant increased or decreased relative abundance (Anders and Huber 2010; Love et al. 2014) in response to phage treatment. Plots at 3 DPT and 30 DPT were separately compared and focused on ASVs that responded to phages in native microbiome background by comparing control plots (no *Pss* or cocktail 5C applied) with plots that received phage treatment only (control vs. cocktail 5C). Plots that received cocktail 5C after *Pss* application and plots that received *Pss* only (*Pss* vs. *Pss* + cocktail 5C) were also separately compared. Only ASVs with mean normalised counts above 100 and 25 were used in differential abundance analysis (DESeq2) of fungi and bacteria, respectively. Probability values reported by DESeq2 were adjusted for multiple testing using the Benjamini–Hochberg (BH) method (Benjamini and Hochberg 1995).

The raw microbiome data can be accessed from the European Nucleotide Archive (project number PRJEB83177). All R scripts used for microbiome data analysis are available at https://doi.org/10.5281/zenodo.14285774.

## 3 Results

**FIGURE 1:**
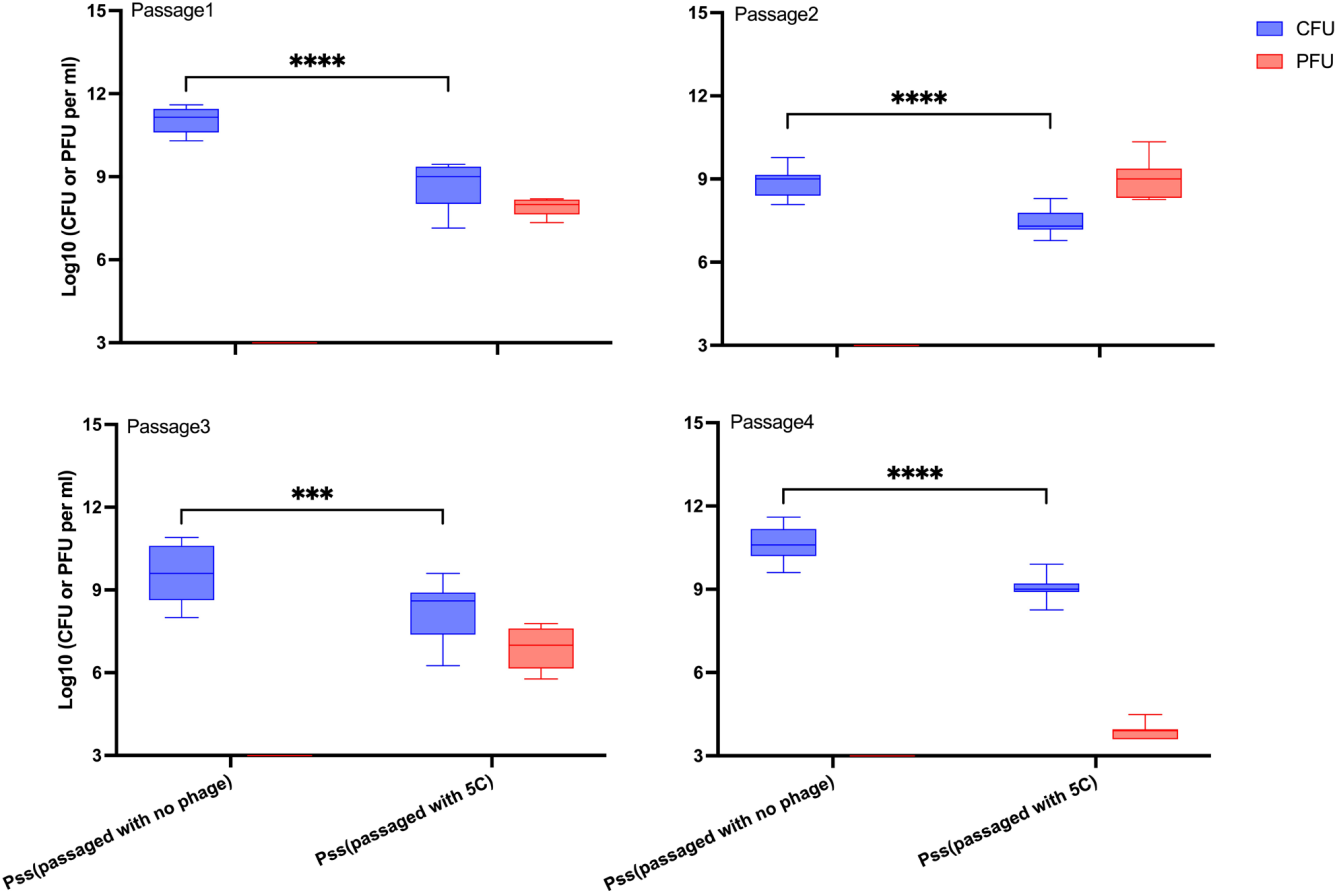
Phage cocktail 5C reduced *Pss* numbers on cherry leaves and remained detectable over time. Population counts of *Pseudomonas syringae* pv. *syringae* strain 9097 (*Pss*) and phage cocktail 5C after passaging four times on detached cherry leaves. Each passage was 72 h long. Three leaves per passage per treatment were spray inoculated with *Pss* or *Pss* and phages. Each boxplot represents three biological and three technical replicates (*n* = 9). Box plots represent colony forming unit (CFU) of bacterial population per leaf (blue) the plaque forming unit (PFU) of phage population on each leaf (red). Significant differences (Tukey) between treatment groups are denoted as ***< 0.001, ****< 0.0001.

Phage resistant *Pss* could not be detected on detached cherry leaves after exposure to phages. A previous study has shown *Pss* evolves phage resistance under pressure of infection in in vitro experiments (Rabiey et al. 2020). To test whether this also occurs in plant tissue, a coevolutionary passaging experiment on cherry leaves was performed. Detached cherry leaves were used for a four‐passage experiment and were inoculated with *Pss*, either alone or with five phages, which were applied either individually or in a cocktail 5C. At each passage for each treatment, *Pss* population levels were measured (Figure 1). Initially, *Pss* populations without phage were extremely high, around 10^11^ CFU mL^−1^, and then remained at between 10^8^ and 10^10^ CFU mL^−1^ over each passage until the end of the experiment. However, when *Pss* populations were exposed to the phage cocktail 5C, the CFU was significantly lower than the control populations, decreasing to just under 10^8^ CFU mL^−1^ during the first passage and remaining at this level till the end of the experiment. Phage populations decreased over each passage, with levels dropping from 10^8^ PFU mL^−1^ in the first passage to 10^4^ PFU mL^−1^ by the fourth passage. These trends were similar across all five individual phage treatments.

**FIGURE 2:**
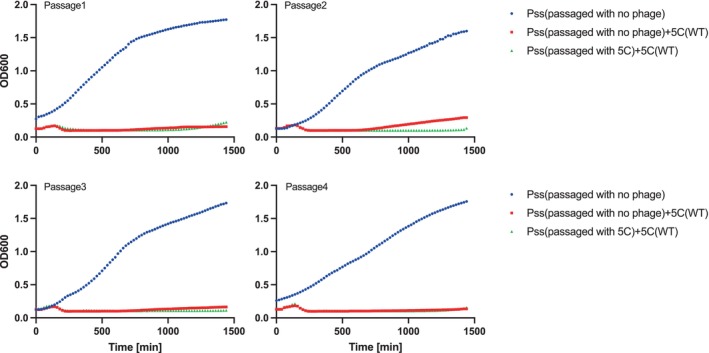
No detectable resistance to phage cocktail 5C was observed for *Pss* isolates recovered from cherry leaves. In vitro killing curve of phage cocktail 5C at multiplicity of infection of 0.01 on *Pseudomonas syringae* pv. *syringae* strain 9097 (*Pss*) isolates collected at each passage, during a *Pss*‐cocktail 5C coevolution on detached cherry leaves. *Pss* recovered from leaf samples with no phage treatment and used as a growth control (blue line); the same cells but challenged with wildtype 5C (red line); recovered from samples with phage treatment in the field and challenged with wildtype 5C (green line). Each line is the mean of three biological and three technical replicates. Statistical analysis is shown in Table S1.

A killing curve assay was performed to investigate whether ancestral wildtype phages were still able to infect coevolved *Pss* isolates collected at different passages (Figures 2 and S2, Table S1). The growth of all *Pss* isolates was prevented by ancestral phage application, suggesting that no detectable resistance emerged in *Pss* isolates following phage application *in planta*.

**FIGURE 3:**
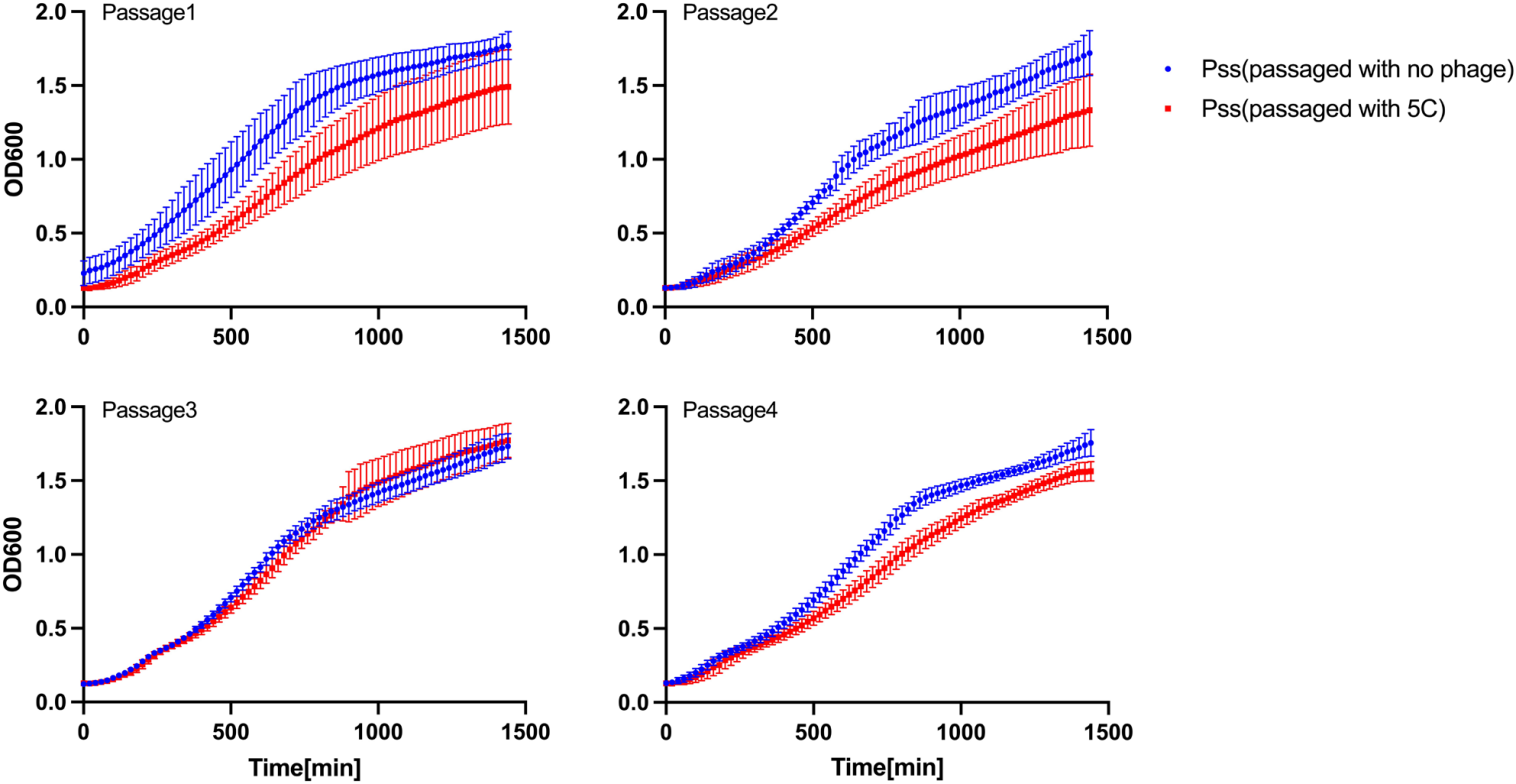
Phage‐treated *Pss* isolates exhibited a slower or similar growth pattern to the *Pss* only treatment that was passaged during the experiment. In vitro growth curve of re‐isolated *Pseudomonas syringae* pv. *syringae* strain 9097 (*Pss*) after passaging with no phage or with phage cocktail 5C on detached cherry leaves. *Pss* isolates were collected at each passage and three colonies were tested per leaf per treatment. Each line is the mean of three biological and three technical replicates. Error bars represent standard deviation. Statistical analysis is shown in Table S1.

Given the apparent lack of phage resistance, we predicted there would likely be no obvious impact on fitness (observed as changes in growth compared to the wildtype) of *Pss* isolates taken from the passages. In vitro growth curve assays were conducted on *Pss* isolates from each passage (with and without phage treatments) (Figures 3 and S3, Table S1). *Pss* coevolved with cocktail 5C grew significantly slower than *Pss* passaged without phage in passages 1, 2 and 4 (Table S1). However, *Pss* coevolved with individual phages had similar growth patterns to the *Pss* coevolved with no phage (Figure 3).

To determine whether phage treatments over the passages had any impact on *Pss* growth *in planta*, isolates were infiltrated into cherry leaves and bacteria recovered and enumerated 7 days post‐inoculation (Figure S4). No significant changes in growth were observed for any of the strains co‐evolved with individual phages or cocktail 5C. This suggests that the application of individual phage or cocktail 5C in the *in planta* coevolutionary experiment did not cause changes in *Pss* strains that affected their growth in plants.

### 3.1 Phages Survived and Multiplied on Cherry Leaves and Shoots in a Cherry Orchard

**FIGURE 4:**
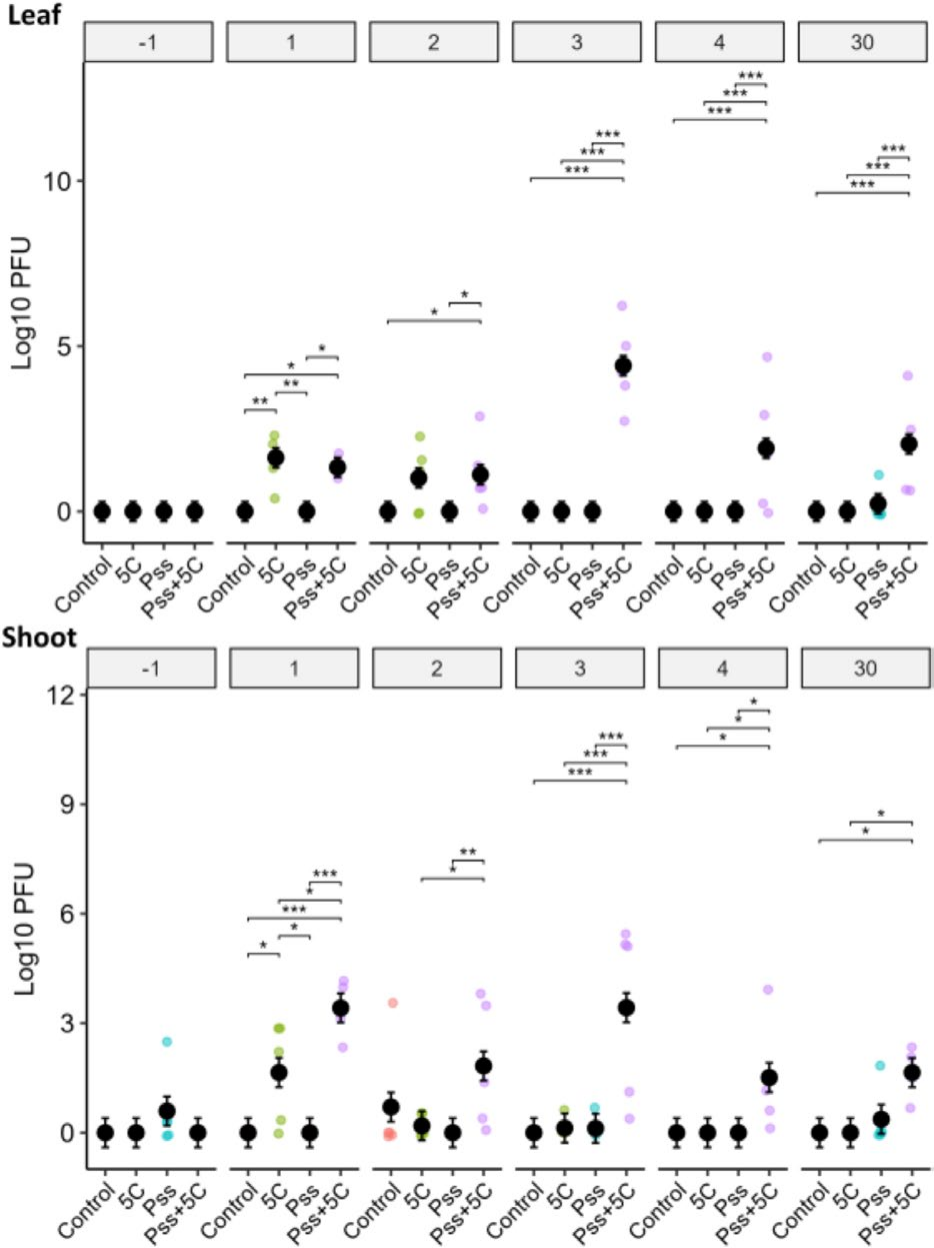
Phage cocktail 5C remained detectable 30‐day post treatment, but only when the host (*Pss*) was present. Population counts of the phage plaque forming units (PFU) on cherry leaves and shoots from the field experiment were calculated (*n* = 5). Samples were obtained 1 day before *Pss* and phage application (‘−1’), and at Day 1 (‘1’), Day 2 (‘2’), Day 3 (‘3’), Day 4 (‘4’) and Day 30 (‘30’) following the *Pss* and phage application. PFU counts are presented as log10 transformed raw data (coloured points, where there are values above zero) and estimated marginal means +/− SEM (black circles). Significant differences calculated via Tukey–Kramer HSD test between treatment groups within the same time point are denoted as *< 0.05, **< 0.01, ***< 0.001. Comparisons of PFU counts within the same treatment group across different time points are shown in Figure S1. The number of bacterial CFU detected in the same samples is shown in Figures S6 and S7.

To use phages as a biocontrol method to treat bacterial diseases, it is essential to assess phage viability, survival and safety within their natural environment. To test this, a field trial in a cherry orchard was conducted, applying four treatments: PBS (control), *Pss*, cocktail 5C and *Pss* and cocktail 5C. Phages from cocktail 5C, without *Pss* host, were detected on leaves and shoots at 1 and 2 DPT (Figures 4 and S5), but not after 3 DPT on leaves and after four DPT on shoots. Where leaves or shoots were treated with *Pss* and cocktail 5C, PFUs were detected at 1, 2, 3, 4 and 30 DPT (Figure 4). There was a notable peak in the PFU count at three DPT in both leaf and shoot samples (Figure S5) where *Pss* and the cocktail 5C were co‐applied. These data indicate that phage can only survive and multiply when in the presence of the bacterial host within the orchard environment. A small number of plaques were isolated from control and *Pss* treated leaves and shoots. The observed plaques were much smaller (pin size) than the expected size of the phages in cocktail 5C (between 5 and 10 mm), suggesting they were produced by phages pre‐existing in the orchard prior to this experiment.

Application of cocktail 5C reduced bacterial numbers in one DPT on leaves and two DPT on shoots (Figures S6 and S7, Table S1), but not over 30 days, where numbers increased (Figures S6 and S7). CFUs for all treatments on both shoots and leaves were significantly higher by 30 DPT compared to 1 DPT.

### 3.2 Exposure to Cocktail 5C Under Orchard Conditions Did Not Impact *Pss* Growth or Phage Sensitivity

**FIGURE 5:**
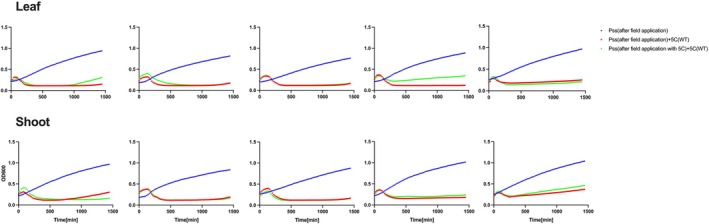
No resistance to phage cocktail 5C was detected in the *Pss* population collected after the field application on cherry leaves and shoots. In vitro kill curve of *Pss* 9097 after the field application with cocktail 5C at multiplicity of infection of 0.01. Five graphs are shown, indicating performance of *Pss* re‐isolated from each tissue at (graphs, left to right) 1‐, 2‐, 3‐, 4‐ and 30‐day post treatment (DPT): Recovered from samples with no phage treatment and used as a growth control (blue line); the same cells but challenged with wildtype 5C (red line); recovered from samples with phage treatment in the field and challenged with wildtype 5C (green line). *Pss* isolations were collected from five leaves and shoots. Each line is the mean of five biological and three technical replicates. Statistical analysis is shown in Table S1.

One key consideration for the use of phages as therapy against bacterial plant diseases is to ensure that the treatment is robust, that is, the use of phages does not drive the evolution of new genotypes of the pathogen. This might be detected as changes in fitness (such as growth) or phage‐sensitivity (emergence of phage resistant mutants). A killing curve assay was performed using the wildtype phage cocktail 5C against *Pss* isolates collected at different time points from the field samples. This showed that cocktail 5C was still able to kill all the *Pss* isolates taken from both leaves and shoots from *Pss* only and *Pss* and cocktail 5C treated samples (Figure 5, Table S1). The growth rates of isolates treated with *Pss* and cocktail 5C exhibited a similar growth pattern (Figure S8, Table S1) to those treated with *Pss* only. Each bacterial isolate from the field treatments, where *Pss* was applied, was confirmed to be *Pss* through PCR with *Pss*‐specific primers. These observations suggest that the application of the phage cocktail 5C did not lead to discernible changes in bacterial fitness or phage sensitivity.

### 3.3 Application of Cocktail 5C in the Orchard Did Not Affect the Quantity of Cherry Leaf Microbiome

It would be hoped that phage specificity towards the target pathogen would not have an impact on the wider leaf microbial community and thus not affect any tree microflora that could be supporting plant health. To determine if phage application might have any indirect impacts, the abundance and diversity of the cherry leaf microbiome were analysed. Based on a preliminary experiment assessing discernible changes, leaf samples were taken at 3 and 30 DPT. The quantity of total bacterial (16S copy number) and fungal (ITS copy number) microbiomes on cherry leaves was not affected by the application of cocktail 5C, *Pss* or their combination (Figure S9). The summary of sequencing reads per sample or per ASV is provided in Tables S2 and S3. In total, there were 565 bacterial ASVs and 699 fungal ASVs. The two most common bacterial classes were Alpha‐ and Beta‐proteobacteria (Figure S11). The most common fungal classes were Taphrinomycetes, Tremellomycetes and Deuteromycetes (Figure S11).

### 3.4 Application of Cocktail 5C Leads to Small Changes in Cherry Leaf Microbiome Alpha Diversity, but Is Dependent on *Pss* Presence and Sampling Time

**FIGURE 6:**
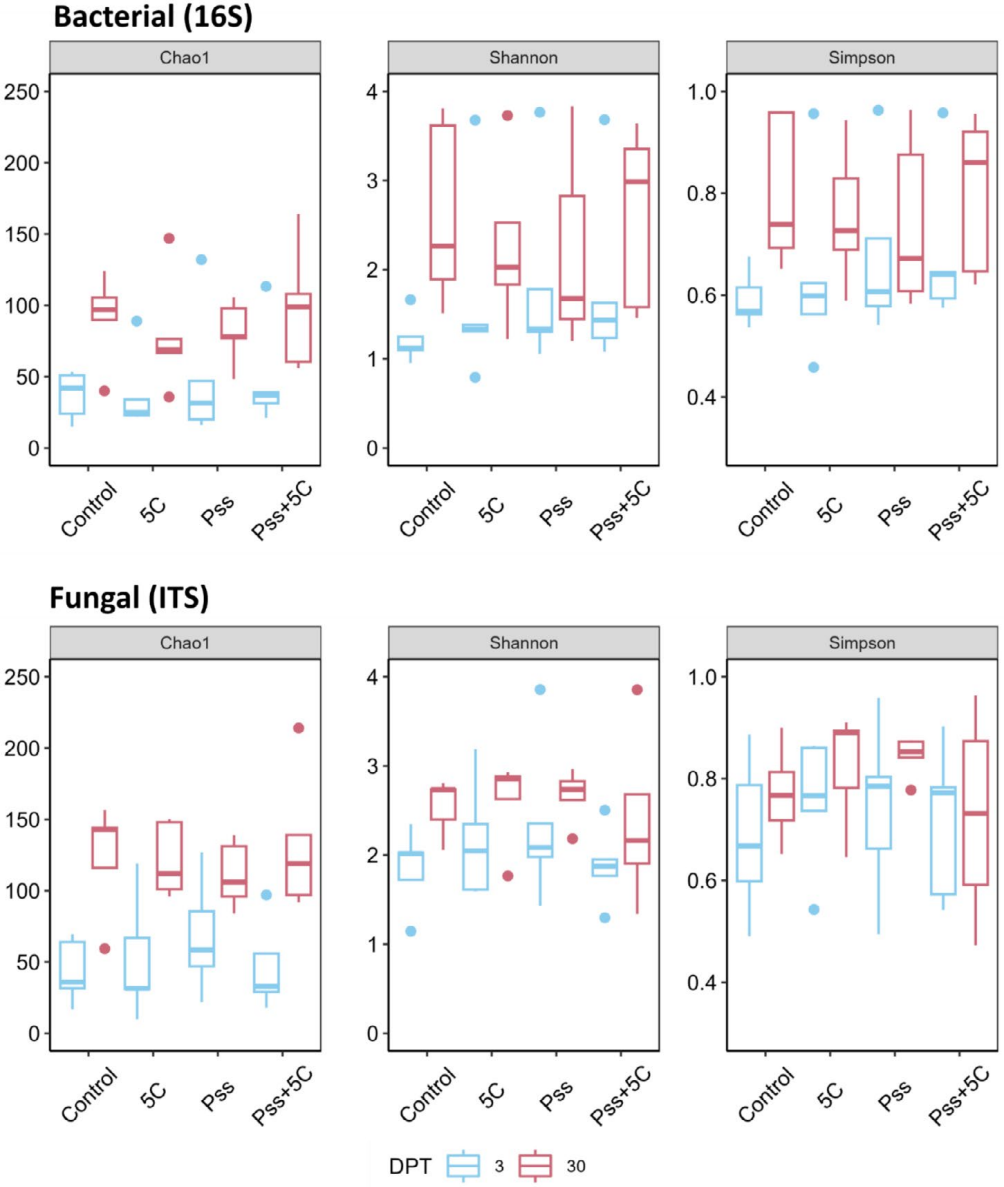
Bacterial and fungal alpha diversity indices from cherry leaves sampled at 3‐ and 30‐day post treatment (DPT). Treatments: *Pss* 9097, phage cocktail 5C, *Pss* + phage cocktail 5C and control (*n* = 5). Chao1 measure: Estimated total number of species per sample, Shannon's index: Within sample community diversity (higher index indicates higher diversity); Simpson index: Community evenness (higher index indicates a more even community).

To determine if the application of cocktail 5C affected microbial diversity, alpha diversity was analysed. Bacterial Chao1 (estimate of the total number of species) was most affected by block (df = 4, *F* = 4.47, *p* < 0.001) and sampling time (df = 1, *F* = 33.21, *p* < 0.001), with more bacterial taxa found at 30 DPT compared to 3 DPT. There was a small effect of cocktail 5C on Chao1 when phage was co‐applied with *Pss* (df = 1, *F* = 0.38, *p* = 0.041). The application of cocktail 5C increased the estimate of the total number of bacterial species (Chao1)when co‐applied with *Pss* (compared to *Pss* only) but decreased Chao1 when applied alone (compared to PBS) at both sampling time points (Figure 6). Shannon's diversity of bacteria was most affected by sampling time (df = 1, *F* = 13.49, *p* = 0.002) and block (df = 4, *F* = 2.23, *p* = 0.026), with higher diversity observed at 30 DPT compared to 3 DPT (Figure 6). Treatment with cocktail 5C affected Shannon's diversity of bacteria, dependent on *Pss* co‐application and time (df = 1, *F* = 1.87, *p* = 0.001) (Figure 6). Simpson's evenness of bacteria was also significantly affected by sampling time (df = 1, *F* = 12.19, *p* < 0.001). A higher evenness was seen at 30 DPT, and there was a significant interaction between cocktail 5C, *Pss* co‐application, and sampling time (df = 1, *F* = 1.35, *p* < 0.001).

Fungal Chao1 was significantly affected by sampling time (df = 1, *F* = 43.55, *p* < 0.001), with more fungal taxa found at 30 DPT compared to 3 DPT. Surprisingly, there was a small but significant effect of the interaction of cocktail 5C, co‐application with *Pss* and sampling time (df = 1, *F* = 0.90, *p* = 0.003). As for bacteria, the application of cocktail 5C slightly increased the total number of fungal species when co‐applied with *Pss* compared to *Pss* only treated plots, but only at 30 DPT. Shannon's diversity of fungal microbiomes was affected by sampling time (df = 1, *F* = 10.32, *p* = 0.001). Treatment with cocktail 5C and *Pss* affected the diversity of fungal microbiomes (df = 1, *F* = 3.6, *p* < 0.001) (Figure 6). This might indicate that adding phages in a high *Pss* pressure scenario results in decreased fungal diversity. Evenness of fungal microbiomes was not affected by sampling time or block. The two‐way interaction of the application of cocktail 5C with *Pss* (df = 1, *F* = 2.12, *p* = 0.007) and the three‐way interaction of this with sampling time (df = 1, *F* = 0.54, *p* = 0.016) had a small but significant effect on the evenness of fungal communities. This suggests that the application of cocktail 5C affected fungal evenness differently depending on the co‐application of *Pss* and sampling time.

Overall bacterial beta diversity (Bray–Curtis dissimilarity) was not affected by block, sampling time or any of the tested cocktail 5C factorial combinations (Figure S12). The effect of cocktail 5C on the fungal composition was significant but very small (as a main effect, df = 1, *R* ^2^ = 0.029, *F* = 1.30, *p* = 0.006), the separation of cocktail 5C treated/untreated samples on the fungal NMDS plot was not obvious (Figure S12). Application of cocktail 5C had a significant effect on the composition of bacterial communities represented by PCs PC14 and PC15, which accounted for 2% and 1.8% of total variance, respectively. Also, the application of cocktail 5C had a small but significant effect on fungal PC6 and PC9, representing 2.9% and 2.6% of the total variance (the co‐occurrence networks of bacterial and fungal ASVs for the mentioned PCs are shown in Figures S13 and S14).

### 3.5 Application of 5C Altered Relative Abundance of Specific Bacterial and Fungal Taxa

Differentially abundant ASVs are listed in Tables 1, 2, 3. Bacterial abundance of two taxa increased, and one taxon decreased on leaves treated with cocktail 5C compared to PBS treatments at three DPT. Relative abundance of ASV28 (*Pseudomonas*) increased when 5C was applied without *Pss* (5C vs. Control). ASV28 shared 100% identity with *Pseudomonas cerasi* (NR_146827.1), *P. ficuserectae* (NR_040798.1), *P. syringae* (NR_117820.1) and *P. congelans* (NR_028985.1) type strains. It is possible that this is *P. syringae* or a closely related strain that is not susceptible to 5C, which is utilising nutrients and niche space vacated by a 5C‐susceptible naturally occurring *P. syringae* strain(s) (Table S4). At 30 DPT, only two taxa were increased, and one decreased in samples treated with *Pss* and cocktail 5C compared to *Pss*. The two increased taxa include ASV23, which has a 100% identity with *Pseudomonas graminis* (NR_026395.1) and *P. rhizosphaerae* (NR_029063.1). These suggest the application of cocktail 5C results in an increase in Pseudomonads in the leaf microbiome; it is possible that these Pseudomonads are occupying the niche once habited by *Pss* once killed off by cocktail 5C.

**TABLE 1:** Summary of differential abundance analysis of bacterial (16S) and fungal (ITS) communities in cherry leaf at two sampling time points.

Amplicon	Time (DPT)	ASVs tested	Group 1	Group 2	Total diff. abundant	Increased	Reduced
16S	3	300	5C	Control	9 (17)	2 (4)	7 (13)
			*Pss* + 5C	*Pss*	6 (14)	3 (4)	3 (10)
	30	458	5C	Control	1 (79)	1 (37)	0 (40)
			*Pss* + 5C	*Pss*	3 (92)	2 (53)	1 (39)
ITS	3	451	5C	Control	16 (98)	4 (54)	12 (44)
			*Pss* + 5C	*Pss*	6 (84)	1 (21)	5 (63)
	30	460	5C	Control	7 (96)	3 (45)	4 (51)
			*Pss* + 5C	*Pss*	11 (124)	10 (84)	1 (40)

*Note:* ASVs with non‐zero mean count across the contrasted groups were used in the test (ASVs tested). The total differentially abundant ASVs, and the ASVs that had significantly reduced or increased abundance in the Group 1 compared to Group 2 are shown as ‘*x* (*y*)’. The ‘*x*’ is the number of differentially abundant ASVs with adjusted p‐value below 0.05 and mean count above 25 and 100 for 16 s and ITS, respectively, and ‘*y*’ is the total number of differentially abundant ASVs with adjusted p‐value below 0.05. The number in the brackets are total differential abundance and the number outside of brackets are differential abundance with mean abundance above 50.

**TABLE 2:** Bacterial (16S) and fungal (ITS) ASVs with significantly different abundance between two tested groups at 3‐day post treatment.

Time (DPI)	Amplicon	Group 1	Group 2	ASV	Mean count	Log2 fold change	Adj. *p*	Taxonomic rank
3	16 s	5C	Control	ASV100	80.8	14.8	7.64E‐03	*Faecalibacterium* (*g*)
				ASV28	70.4	24.5	5.72E‐08	*Pseudomonas* (*g*)
				ASV139	187.3	−29.3	1.31E‐09	*Moraxella* (*g*)
				ASV62	140.4	−16.0	2.94E‐03	*Ruminococcus* (*g*)
				ASV159	112.5	−14.0	1.59E‐02	*Paracoccus* (*g*)
				ASV257	92.9	−28.2	3.67E‐09	*Geobacillus* (*g*)
				ASV187	72.5	−28.0	3.67E‐09	*Bacteroides* (*g*)
				ASV27	49.0	−28.7	1.31E‐09	*Acinetobacter* (*g*)
				ASV193	39.7	−26.8	1.60E‐08	*Labilithrix* (*g*)
3	16 s	*Pss* + 5C	*Pss*	ASV166	260.9	28.1	2.42E‐09	*Pseudomonas* (*g*)
				ASV157	96.8	20.7	2.64E‐05	*Lawsonella* (*g*)
				ASV31	74.4	27.9	2.42E‐09	*Novosphingobium* (*g*)
				ASV227	136.9	−21.2	1.80E‐05	*Massilia* (*g*)
				ASV150	93.1	−20.9	2.30E‐05	*Lysobacter* (*g*)
				ASV27	49.0	−28.9	2.02E‐09	*Acinetobacter* (*g*)
3	ITS	5C	Control	ASV15	332.0	22.5	9.51E‐07	*Vishniacozyma victoriae* (*s*)
				ASV20	275.7	10.3	4.36E‐02	*Fusarium* (*g*)
				ASV35	249.5	15.4	1.29E‐03	*Malassezia globosa* (*s*)
				ASV12	147.7	23.7	3.92E‐07	*Tremellales* (o)
				ASV16	455.0	−13.8	2.52E‐03	*Nothophoma* (*g*)
				ASV6	329.6	−11.9	8.63E‐03	*Vishniacozyma carnescens* (*s*)
				ASV209	246.0	−26.1	3.02E‐08	*Fungi* (*k*)
				ASV88	216.1	−23.6	6.05E‐07	Lecanorales (o)
				ASV58	203.6	−17.7	1.75E‐04	*Ogataea polymorpha* (*s*)
				ASV13	193.7	−12.6	1.18E‐02	*Sporidiobolus* sp. (*s*)
				ASV19	141.8	−15.7	1.99E‐04	*Alternaria alternata* (*s*)
				ASV524	134.5	−24.9	1.24E‐07	*Fungi* (*k*)
				ASV147	123.8	−17.4	1.97E‐04	*Aspergillus glaucus* (*s*)
				ASV14	119.8	−11.6	1.22E‐02	*Vishniacozyma tephrensis* (*s*)
				ASV25	114.8	−20.8	6.78E‐06	*Clavariopsis aquatica* (*s*)
				ASV175	102.7	−33.1	5.29E‐13	*Fusarium* (*g*)
3	ITS	*Pss* + 5C	*Pss*	ASV103	214.9	26.5	1.32E‐08	Sordariales (o)
				ASV9	704.6	−34.1	1.17E‐13	*Schizophyllum commune* (*s*)
				ASV7	239.5	−14.0	3.94E‐03	*Monilinia* (*g*)
				ASV72	132.0	−29.4	2.03E‐10	Lecanorales (o)
				ASV25	114.8	−21.2	8.96E‐06	*Clavariopsis aquatica* (*s*)
				ASV175	102.7	−24.6	1.57E‐07	*Fusarium* (*g*)

*Note:* Only ASVs with average read counts above 25 and 100 are shown for bacterial (16S) and fungal (ITS) taxa, respectively. Positive Log2 fold change values indicate higher abundance in Group 1. Taxonomy rank above 80% confidence is shown.

Abbreviations: f: family, *g*: genus, o: order, *s*: species.

**TABLE 3:** Bacterial (16S) and fungal (ITS) ASVs with significantly different abundance between two tested groups at 30 days post treatment.

Time (DPI)	Amplicon	Group 1	Group 2	ASV	Mean count	Log2 fold change	Adj. *p*	Taxonomic rank
30	16 s	5C	Control	ASV30	45.2	23.2	2.94E‐06	*Paracoccus* (*g*)
30	16 s	*Pss* + 5C	*Pss*	ASV23	144.2	11.4	2.21E‐02	*Pseudomonas* (*g*)
				ASV40	34.3	21.5	2.68E‐05	*Erwinia* (*g*)
				ASV69	28.2	−14.9	3.87E‐03	*Escherichia/Shigella* (*g*)
30	ITS	5C	Control	ASV51	319.1	25.6	2.76E‐07	Pleosporales (o)
				ASV102	158.6	23.4	1.71E‐06	Fungi (k)
				ASV118	126.2	33.7	2.28E‐12	Fungi (k)
				ASV54	367	−15.3	2.55E‐03	*Scleroconidioma* (*g*)
				ASV78	276.5	−18.1	2.27E‐04	*Clonostachys rosea* (*s*)
				ASV99	254.8	−21.5	9.27E‐06	Sordariomycetes (c)
				ASV113	136	−14.2	5.99E‐03	Trimorphomycetaceae (f)
30	ITS	*Pss* + 5C	*Pss*	ASV54	367.0	28.8	5.13E‐09	*Scleroconidioma* (*g*)
				ASV51	319.1	26.9	3.73E‐08	Pleosporales (o)
				ASV99	254.8	28.8	5.13E‐09	Sordariomycetes (c)
				ASV101	229.8	24.8	5.53E‐07	Dothideomycetes (c)
				ASV81	176.9	25.6	2.37E‐07	Ascomycota (p)
				ASV96	171.6	21.8	1.11E‐05	Dothideomycetes (c)
				ASV102	158.6	25.0	3.90E‐07	Fungi (k)
				ASV63	143.9	24.1	1.16E‐06	*Pestalotiopsis* (*g*)
				ASV113	136.0	28.0	1.32E‐08	Trimorphomycetaceae (f)
				ASV118	126.2	21.5	1.34E‐05	Fungi (k)
				ASV219	101.5	−16.4	8.66E‐04	Basidiomycota (p)

*Note:* Only ASVs with average read counts above 25 and 100 are shown for bacterial (16S) and fungal (ITS) taxa, respectively. Positive Log2 fold change values indicate higher abundance in Group 1.

Abbreviations: P: phylum, o: order, f: family, *g*: genus, *s*: species.

An increase in four and a decrease in 12 different fungal taxa compositions was observed at three DPT in leaves treated with cocktail 5C in comparison to PBS. ASVs with decreased taxa included filamentous plant pathogenic fungi such as *Nothophoma* ASV16, *Alternaria alternata* ASV19 and *Fusarium* ASV175. In comparing the application of *Pss* and cocktail 5C to *Pss* at three DPT, only one taxon increased in abundance (Sordariales ASV103), whereas five taxa decreased, two being potentially pathogenic filamentous fungi, that is, *Monilinia* ASV7 and *Fusarium* ASV175 (Table 2).

## 4 Discussion

This study explored the ecological impacts of bacteriophage biocontrol against the cherry canker pathogen (*Pss*) *in planta*, focusing on the longevity of viable phages, the effects on the target pathogen's fitness (growth) and the implications for the plant leaf microbiome. This included both detached cherry leaves and a full‐scale production orchard, marking the first time such a comprehensive study has been conducted in an orchard setting.

The coevolutionary passaging of five MR phages individually or in a cocktail 5C with *Pss* on detached cherry leaves revealed no evidence of resistance emergence in the *Pss* population (Figure 2). Moreover, no evidence of phage resistant *Pss* was found after applying phages to cherry trees within an orchard setting (Figure 5). This finding aligns with the findings seen in Hernandez and Koskella (2019), as well as broader trends observed in other studies investigating the experimental evolution of bacteria with phages in both plant and animal systems. For instance, Maura et al. (2012), Meaden and Koskella (2017), and De Sordi et al. (2019) have reported similar outcomes, often noting limited or entirely absent evidence of phage resistance evolution in their respective experiments. There are several reasons why phage resistance may not evolve *in planta*. First, mutations that confer phage resistance can often negatively impact bacterial fitness, such as growth, motility and pathogenicity (Bartnik et al. 2022; Liu, Tian, et al. 2022; Wang et al. 2019). Such resistance may thus be fatal within the harsh environment of leaves or tree bark tissue, compared to controlled in vitro conditions seen in the lab. The lack of phage resistance in the coevolution study and the field trial study is likely the reason why there is either none or very little change in growth (in vitro or *in planta*) of the phage‐treated colonies compared to the control colonies (Figures 3, S4 and S8). Only the use of cocktail 5C (but not individual phages) caused small drops in *Pss* fitness over three of the four time points in the passaging experiment when tested in vitro. The stochastic nature of the changing fitness between passages may indicate small evolutionary changes over time in the pathogen. This observation is not unexpected given the bacteria were evolved within the plant environment and not a liquid broth. Reinforcing this premise, no fitness cost was observed for the same *Pss* isolates when infiltrated on cherry leaves. Secondly, leaf or bark tissue provides a much more heterogeneous environment for phage and bacteria interactions, enabling them to fill spatial niches and reducing the likelihood of encountering each other, meaning bacterial lysis by phage may occur at a slower rate. These collective findings suggest a nuanced interplay between phage resistance and environmental contexts, emphasising the importance of considering diverse ecological conditions when studying the evolution of bacterial resistance to phages.

A cocktail of five MR phages and *Pss* 9097 was applied to the leaves and shoots of mature cherry trees within a commercial orchard. Phages were sprayed without protectants but were applied in the evening to minimise the detrimental effects of UV irradiation and heat. Tracking of the applied phage populations over 30 days found that phages could only be detected if co‐applied with their host, *Pss* (Figures 4 and S5). Phages applied without *Pss* could only be detected up to three DPT. This indicates that phages require a significant host population to be present within the leaf and shoot environment in order to persist. Phage persistence is also largely dependent on environmental factors, with UV irradiation, high temperatures and desiccation proven to degrade phage (Iriarte et al. 2007). However, phage survival can be improved using protective formulations, such as with proteins or sugars (Balogh et al. 2003; Iriarte et al. 2007).

The ability of a phage treatment to suppress a pathogen populations is generally higher in vitro than *in planta* (Balogh et al. 2018). In this study, cocktail 5C effectively reduced *Pss* populations under controlled conditions in the coevolutionary passaging experiment (Figures 1 and S1), but was less effective in the orchard (Figures S6 and S7); only significantly decreased *Pss* populations occurred on tree shoots at two DPT. This reduced efficacy in the field could be due to several reasons. First, to ensure establishment, a high *Pss* concentration was used to infect the leaves and shoots, which would not necessarily be seen in natural *Pss* infections. Second, the lack of phage survival within the orchard environment may have prevented them from killing *Pss*. Third, the colony counting quantification method used included dilution plating on *Pss* selective media, which detected *Pss* 9097 but also naturally occurring *Pss* strains not susceptible to cocktail 5C. This was evident as CFU counts increased significantly from Day 1 to Day 30 in PBS only control and cocktail 5C only plots, indicating an increase in natural Pseudomonad populations. Accurate measurement of cocktail 5C efficacy in the field might require strain‐specific quantification methods, such as digital droplet PCR (Morella et al. 2018).

Alongside abundance, phages can alter bacterial community dynamics and structure (Koskella and Brockhurst 2014; Morella et al. 2018; Rodriguez‐Valera et al. 2009) by removing susceptible strains and releasing cellular nutrients to their competitor microbes (Weitz and Wilhelm 2012), increasing overall community richness and density. A ‘kill‐the‐winner’ model has been proposed in marine ecosystems, where the most abundant bacteria are most susceptible to phage predation and are thus kept from dominating the community (Rodriguez‐Valera et al. 2009). In this study, applying cocktail 5C did not affect the total bacterial community size on cherry leaves (Figure S9). However, it did alter alpha diversity and the relative abundance of certain bacterial ASVs, consistent with the ‘kill‐the‐winner’ model (Figure 6 and Tables 1, 2, S2 and S3). Samples at 30 DPT treated with cocktail 5C and *Pss* had increased alpha diversity indices (total number of species, diversity and evenness) compared to those treated with *Pss* only. This increase is attributed to cocktail 5C reducing a large proportion of the susceptible *Pss* population, which would otherwise dominate and reduce alpha diversity. In the natural microbiome without added *Pss*, there was less strain dominance, a lower proportion of cocktail 5C‐susceptible cells and a negligible effect on alpha diversity. Conversely, overall beta diversity was not affected by cocktail 5C application (Figure S12).

The application of cocktail 5C altered the relative abundance of some bacterial ASVs more significantly at 30 DPT compared to three DPT, indicating initial disruption followed by community stabilisation (Table 1, 2, S2 and S3). A similar pattern was observed in the application of phage to treat *P. syringae* in tomatoes, where a diverse phage assembly impacted the bacterial community more at 1‐day than at 7‐day post‐application (Morella et al. 2018). Phages used against potato wilt and soft rot pathogens also altered bacterial alpha diversity in soil (Mousa et al. 2022), whereas no changes were seen with phages against other pathogens (Magar et al. 2024). Similarly, phage therapy targeting *Escherichia coli* in the human gut reduced *E. coli* numbers but did not affect overall microbiome diversity (Febvre et al. 2019). These findings suggest that phage therapy's impact on microbiomes is context‐specific, influenced by target dominance, competition and microbiome complexity.

The application of cocktail 5C did not change the overall size of the fungal microbiome on cherry leaves (Figure S9). The relative abundance of several basidiomycetous yeasts from the *Vishniacozyma* genus increased, which correlates with reduced disease indices and increased beneficial fungi in wheat (Vujanovic 2021). Notably, *V. victoriae* (ASV15), which increased in abundance, is known to control *Botrytis cinerea* on kiwifruit (Nian et al. 2023). Several potential pathogenic fungi, such as *Nothophoma*, *Fusarium*, *Monilinia* and *Alternaria alternata* , decreased in abundance at 3 DPT. Cocktail 5C application appears to temporarily increase beneficial fungi and reduce potential pathogens. While direct effects of cocktail 5C on fungi are possible, it is more likely that phage‐induced bacterial community changes indirectly affected fungal communities due to their close ecological interactions.

The 16S/ITS amplicon sequencing approach used in this study is well‐established in microbiome research, offering reliable analysis pipelines and taxonomy databases (Liu et al. 2021; Zhang et al. 2023). However, it has limitations, including reduced taxonomic resolution for bacteria, PCR bias, and the impact of sequencing depth on results (Boshuizen and te Beest 2023; Notario et al. 2023; Ramakodi 2021). These limitations and the context‐dependent interactions between phages and plant microbiomes suggest the need for further research to fully understand the effects of phage cocktails like 5C on plant phyllosphere communities. Long‐read sequencing could improve taxonomic resolution (Notario et al. 2023). Future studies should also focus on the functional impacts of phages on microbiomes using approaches like shotgun metagenomics, meta‐transcriptomics and multi‐omics (Burz et al. 2023; Calle 2019; Liu, Hernandez‐Morales, et al. 2022; Puig‐Castellví et al. 2023; Shakya et al. 2019), as well as the effects of phage treatments on plant endospheres and community resilience to stress.

In summary, this study highlights that the application of phage cocktails to cherry leaves can effectively reduce cherry pathogen abundance on the host plant. Moreover, small changes in the bacterial and fungal microbiome constituents correlate with the reduction in the pathogen. Microbial community analysis suggests the niche vacated by *Pss* 9097 is filled by other microbes, some of which could be directly or indirectly beneficial to plant health, for example, by creating a potential barrier against pathogen ingress or via competitive/antagonistic interactions.

## Author Contributions

**Matevz Papp‐Rupar:** methodology, conceptualization, formal analysis, writing – original draft, writing – review and editing, investigation, validation, resources, funding acquisition. **Emily R. Grace:** methodology, validation, formal analysis, visualization, writing – review and editing, writing – original draft, investigation. **Naina Korotania:** methodology, data curation, formal analysis, validation, investigation, writing – review and editing. **Maria‐Laura Ciusa:** methodology, data curation, formal analysis, validation, investigation, writing – review and editing. **Robert W. Jackson:** conceptualization, methodology, supervision, formal analysis, investigation, validation, funding acquisition, visualization, project administration, resources, writing – original draft, writing – review and editing. **Mojgan Rabiey:** conceptualization, methodology, data curation, supervision, resources, formal analysis, project administration, validation, visualization, writing – review and editing, writing – original draft, funding acquisition, investigation.

## Conflicts of Interest

The authors declare no conflicts of interest.

## Supporting information


Data S1.

Table S1.

## Data Availability

The datasets supporting the conclusions of this article are included within the article and its Supporting Information.
