# Supplementary material for: Impact of Phage Therapy on Pseudomonas syringae pv. syringae and Plant Microbiome Dynamics Through Coevolution and Field Experiments
Source: Environ Microbiol. 2025 Mar 12;27(3):e70076. doi: 10.1111/1462-2920.70076 (PMC11903928; doi:10.1111/1462-2920.70076)
Supplement: Supplementary file 1 — Data S1. [file EMI-27-e70076-s001.docx]

APPENDIX


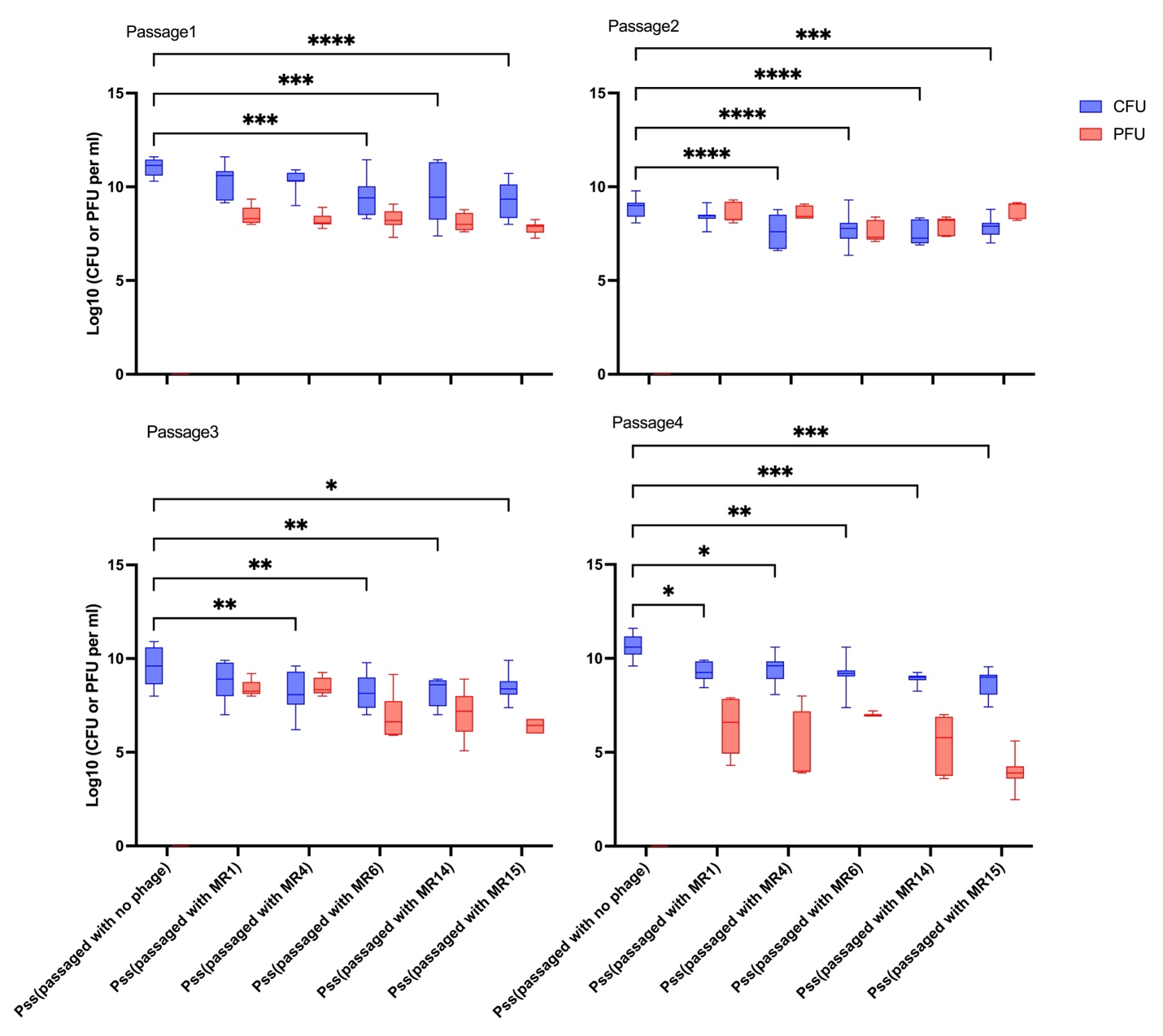


**Figure S1. MR phages remained stable during passaging on cherry leaves and reduced *Pss* population.** Population counts of *Pseudomonas syringae* pv. *syringae* strain 9097 (*Pss*) and phage MR1, MR4, MR6, MR14 and MR15 after passaging four times on detached cherry leaves. Each passage was 72h. Three leaves per passage per treatment were passaged with *Pss* or *Pss* and phages. Box plot represents three biological and three technical replicates (n=9). Box plots represent colony forming unit (CFU) of bacterial population or plaque forming unit (PFU) of phage population per leaf. Significant differences (Tukey) between treatment groups are denoted as ‘*’ <0.05, ‘**’<0.01, ‘***’<0.001, ‘****’<0.0001.


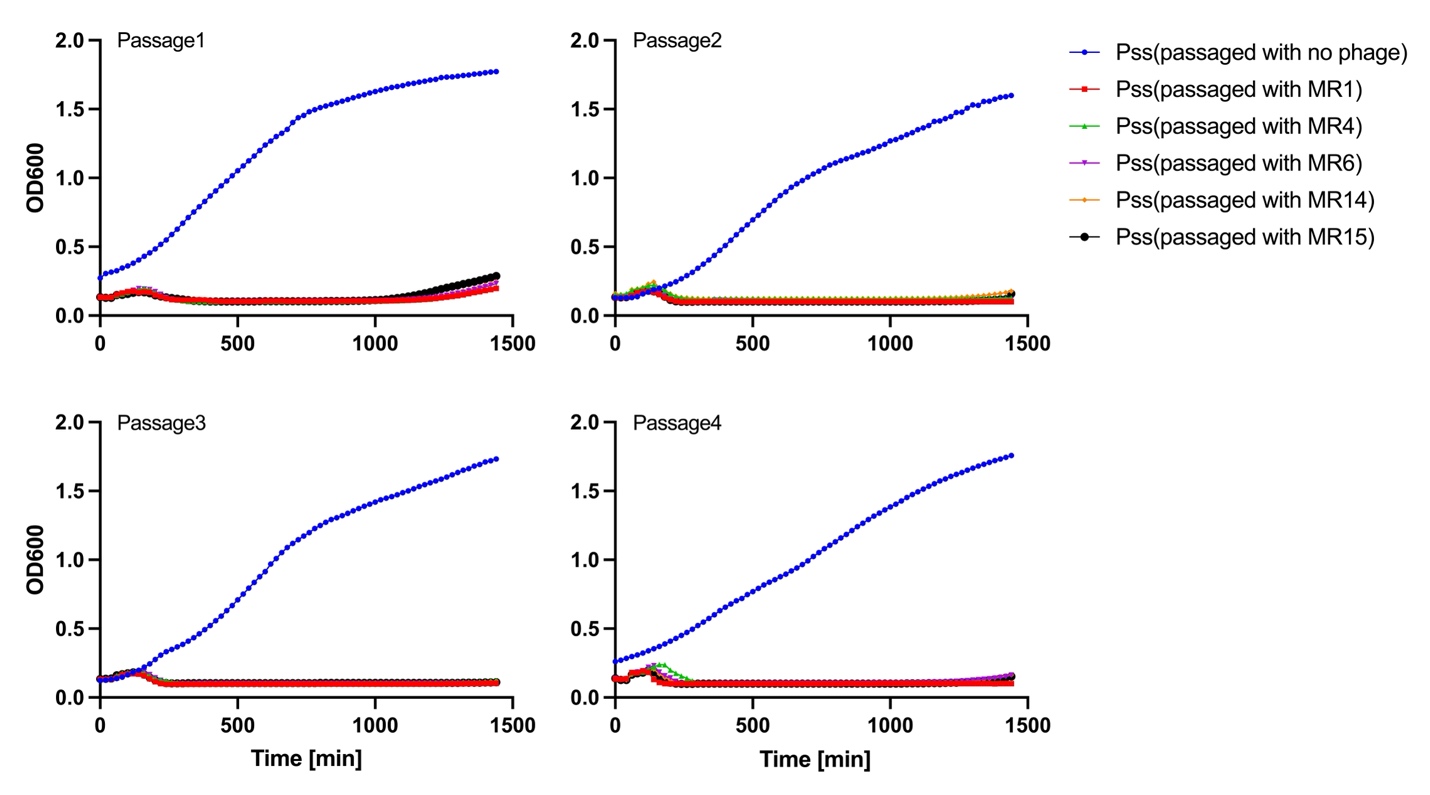


**Figure S2.** **No resistance emerged to MR phages, in the *Pss* population, after passaging on cherry leaves.** *In vitro* killing curve of phage MR1, MR4, MR6, MR14 and MR15 at multiplicity of infection of 0.01 on *Pss* 9097 isolates collected at each passage, during a *Pss*-phage coevolution on detached cherry leaves**.** Each line is the mean of three biological and three technical replicates.


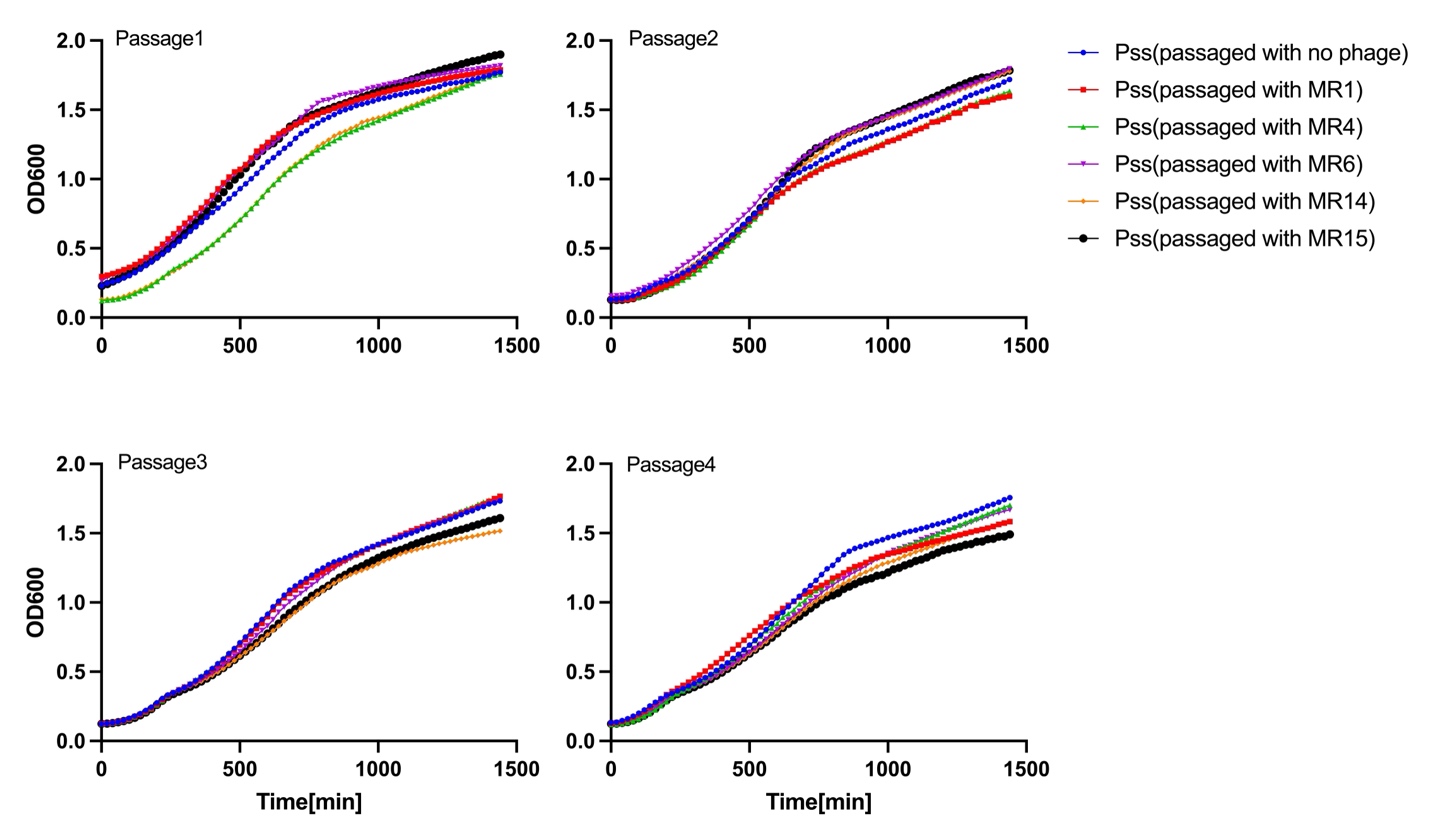


**Figure S3. *Pss* isolates exhibited a similar growth pattern to the *Pss* only treatment that was passaged during the experiment.** *In vitro* growth curve of *Pss* 9097 after passaging with phage MR1, MR4, MR6, MR14 and MR15 on detached cherry leaves. *Pss* population was collected at each passage and 3 colonies were tested per leaf per treatment. Each line is the mean of three biological and three technical replicates

**Figure S4. Coevolution of *Pss* with MR phages did not impact *Pss* fitness (growth) on cherry leaves.** Population counts of *Pss* 9097 isolates collected at each passage, during the *Pss*-phage coevolution on detached cherry leaves**.** *Pss* isolates grown with no phage, phage MR1, MR4, MR6, MR14, MR15 and cocktail 5C collected at passage 1 to 4, from cherry leaves coevolution experiment, were infiltrated into cherry cultivar Sweetheart leaves (three biological and three technical replicates). A mock inoculation with PBS caused no symptom development or recovered bacterial colonies (data not shown). No significant differences were observed (ANOVA Tukey analysis).


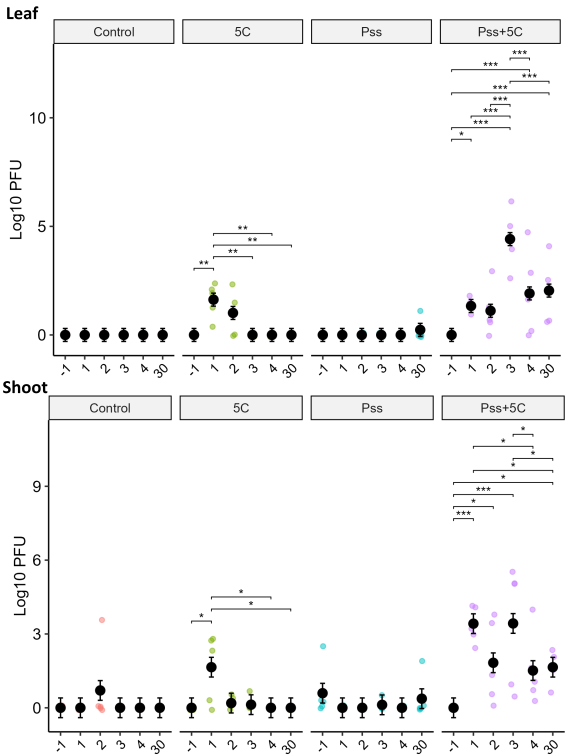


**Figure S5. Phage cocktail 5C remained detectable 30 day post treatment, but only when the *Pss* bacterial host was present.** Population counts of phage plaque forming units (PFU) on cherry leaves and shoots in the field experiment. Samples were obtained 1 day before *Pss* and phage application (‘-1’), and at day 1 (‘1’), day 2 (‘2’), day 3 (‘3’), day 4 (‘4’), and day 30 (‘30’) following the *Pss* and phage application (n = 5). PFU counts are presented as log10 transformed raw data (coloured points) and estimated marginal means +/- standard errors of treatments (x-axis) at different days post treatment. Significant differences (Tukey) between different time points within the same treatment group are denoted as ‘*’ <0.05, ‘**’<0.01, ‘***’<0.001. The number of bacterial CFU detected in the same samples is shown in Figure S7 and Figure S8.


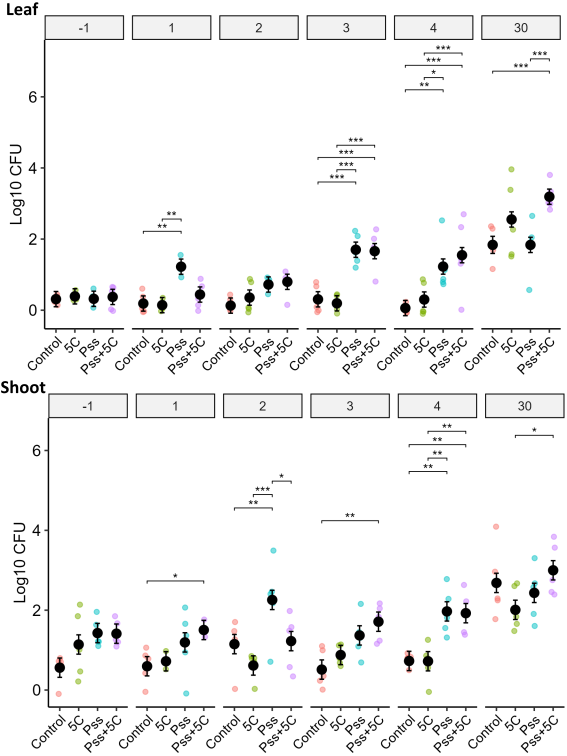


**Figure** **S6. Population count of the bacterial forming units (CFU) detected on cherry leaves and shoots** **in the field experiment.** Samples were obtained 1 day before *Pss* and phage application (‘-1’), and at day 1 (‘1’), day 2 (‘2’), day 3 (‘3’), day 4 (‘4’), and day 30 (‘30’) following the *Pss* and phage application (n = 5). CFU counts are presented as log10 transformed raw data (coloured points) and estimated marginal means +/- standard errors of treatments (x-axis) at different days post treatment. Significant differences (Tukey) between treatment groups within the same time point are denoted as ‘*’ <0.05, ‘**’<0.01, ‘***’<0.001. Comparisons of PFU counts within the same treatment group across different time points are shown in Figure S1. The number of bacterial CFU detected in the same samples is shown in Figure S2 and Figure S3.


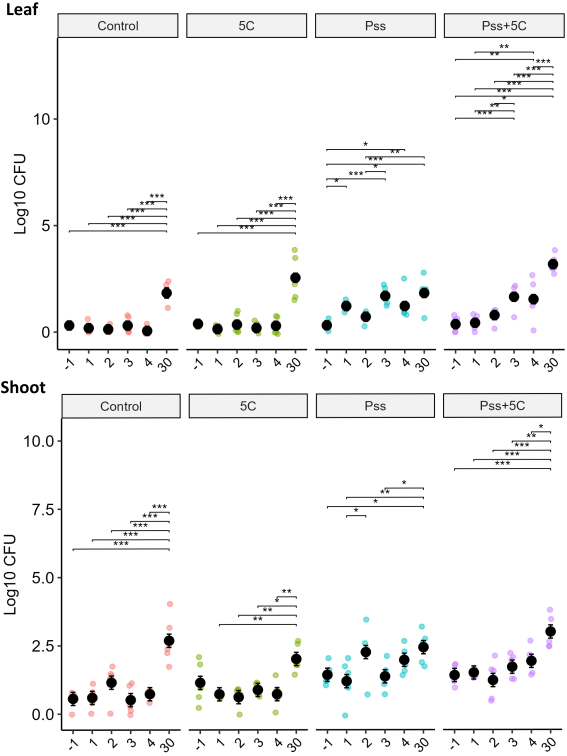


**Figure S7. Population count of the bacterial forming units (CFU) detected on cherry leaves and shoots** **in the field experiment.** Samples were obtained 1 day before *Pss* and phage application (‘-1’), and at day 1 (‘1’), day 2 (‘2’), day 3 (‘3’), day 4 (‘4’), and day 30 (‘30’) following the *Pss* and phage application (n = 5). CFU counts are presented as log10 transformed raw data (coloured points) and estimated marginal means +/- standard errors of treatments (x-axis) at different days post treatment. Significant differences (Tukey) between time points within the same treatment group are denoted as ‘*’ <0.05, ‘**’<0.01, ‘***’<0.001.


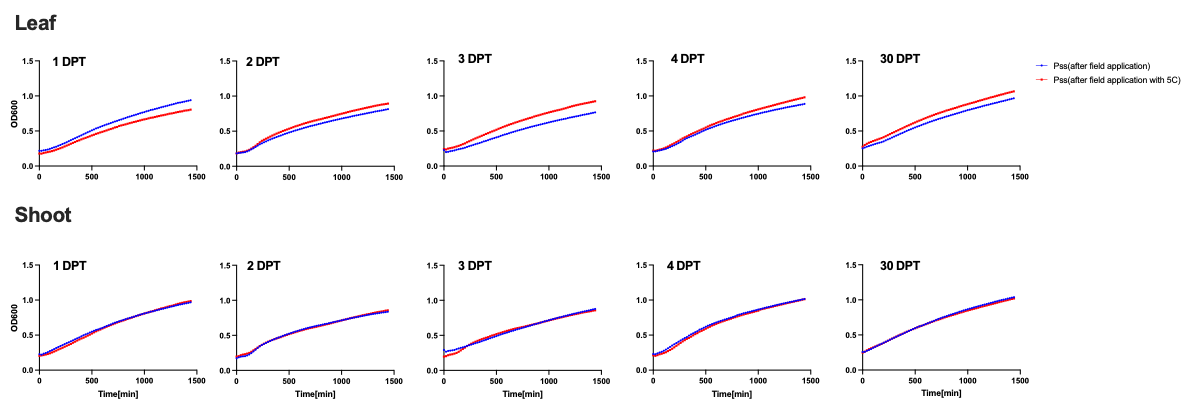


**Figure S8. Growth curve assays of *Pss* re-isolated from leaves and shoots with and without phage 5C application.** *In vitro* growth curve of *Pss* 9097 collected after the field application. *Pss* isolates were collected from no phage and phage cocktail 5C treated leaves or shoots and growth curves are shown in graphs (from left to right) at 1-, 2-, 3-, 4-, and 30-day post treatment (DPT) in the field experiment from 5 leaves. Each line is the mean of five biological and three technical replicates. Statistical analysis is shown in Table S1.


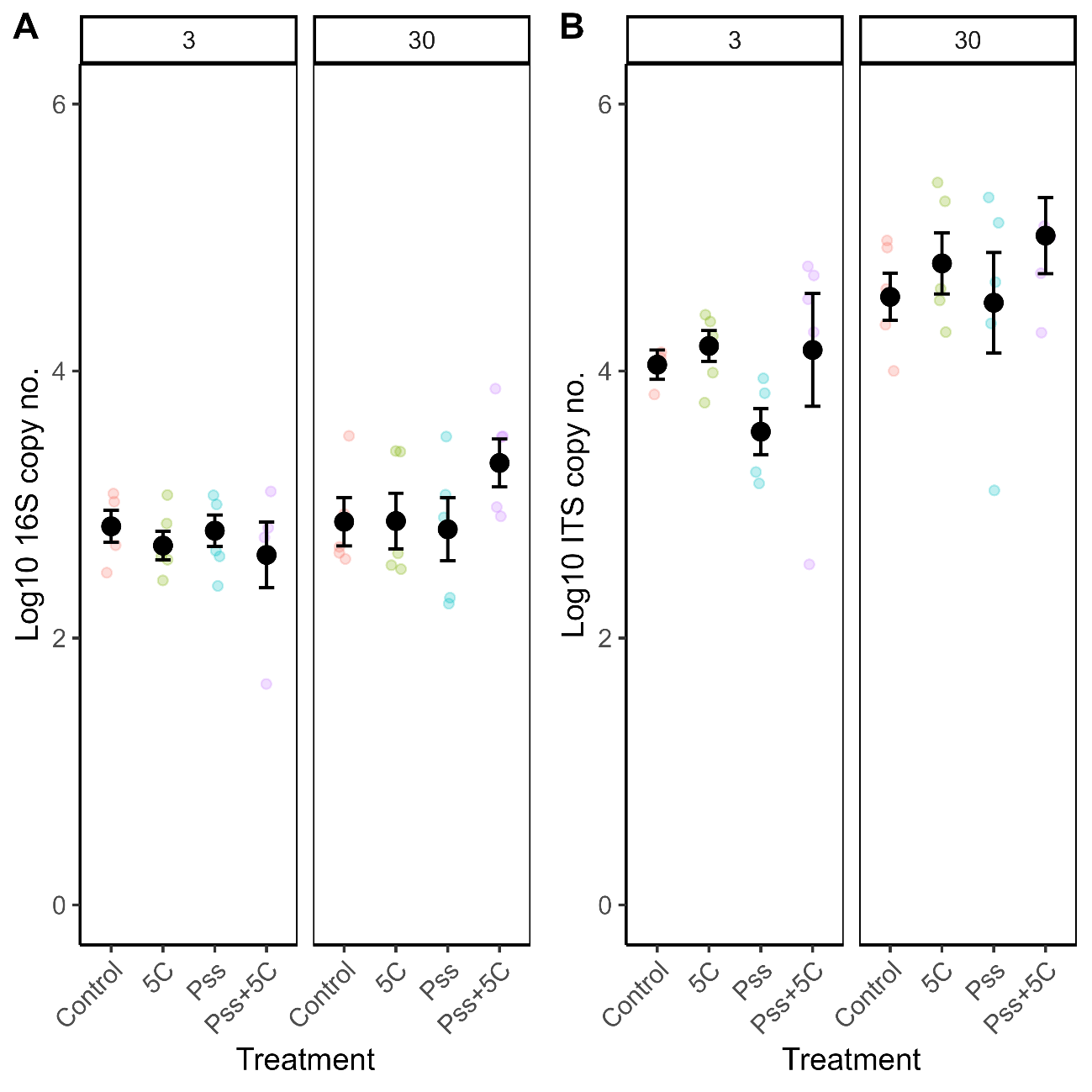


**Figure S9: The total size of bacterial (A) and fungal community on cherry leaves at 3 and 30 day post treatment in the field experiment as measured by qPCR**. Mean +/- SEM and raw data (coloured dots) are presented. There were no significant differences between the control and any of the treatment groups at either of the time points.


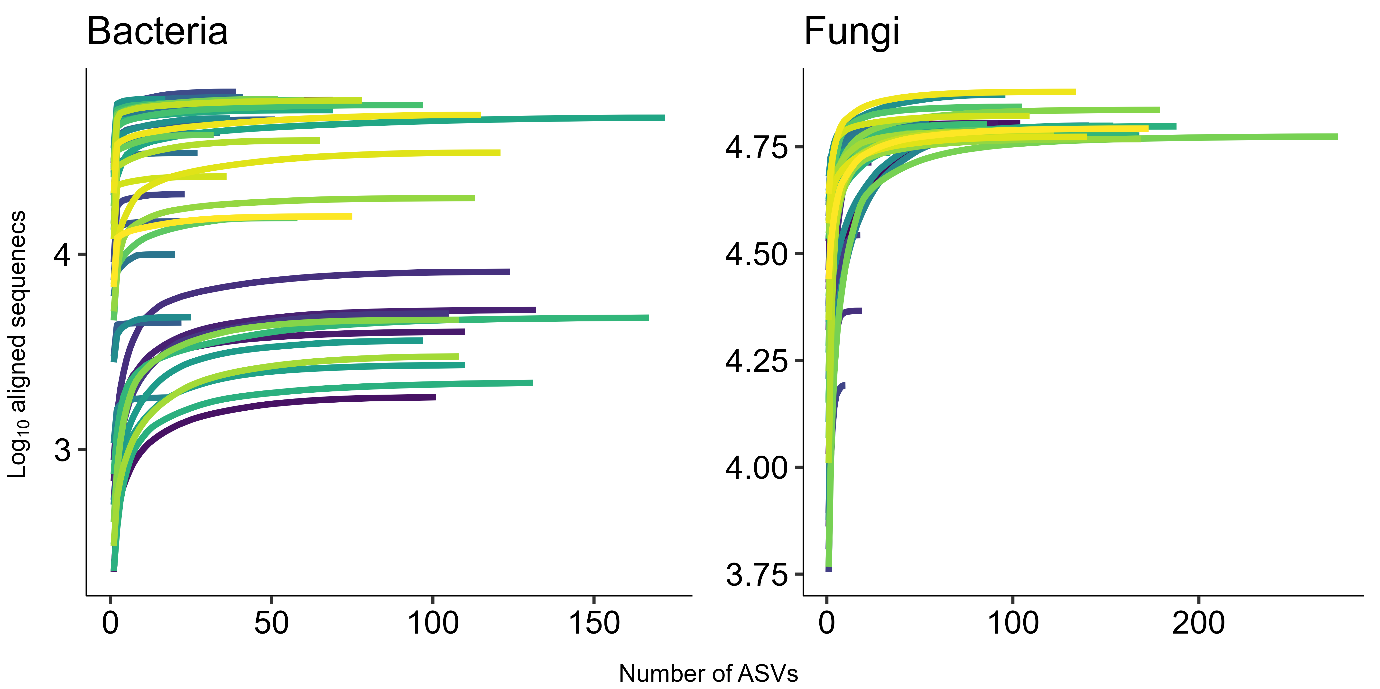


**Figure S10. Rarefaction curves for bacteria (16S) and fungi (ITS) in all the 40 samples across two timepoints. Shown are** 3 and 30 day post treatment results.

**
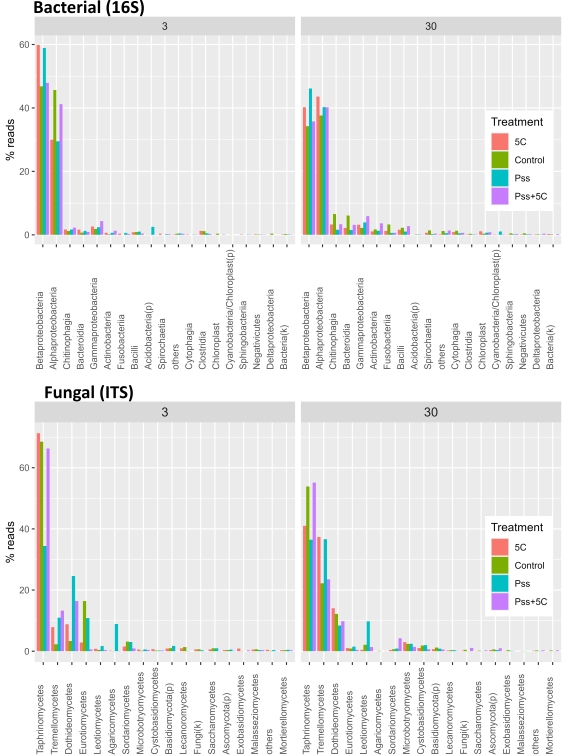
**

**Figure S11**. **Proportion of mapped bacterial (top) and fungal (bottom) reads assigned to each taxonomic class for the 40 leaf samples across two time points.** Shown are data obtained at 3 and 30 days post application and four treatments. Treatments: *Pss* 9097, phage cocktail 5C, *Pss* + phage cocktail 5C and control.


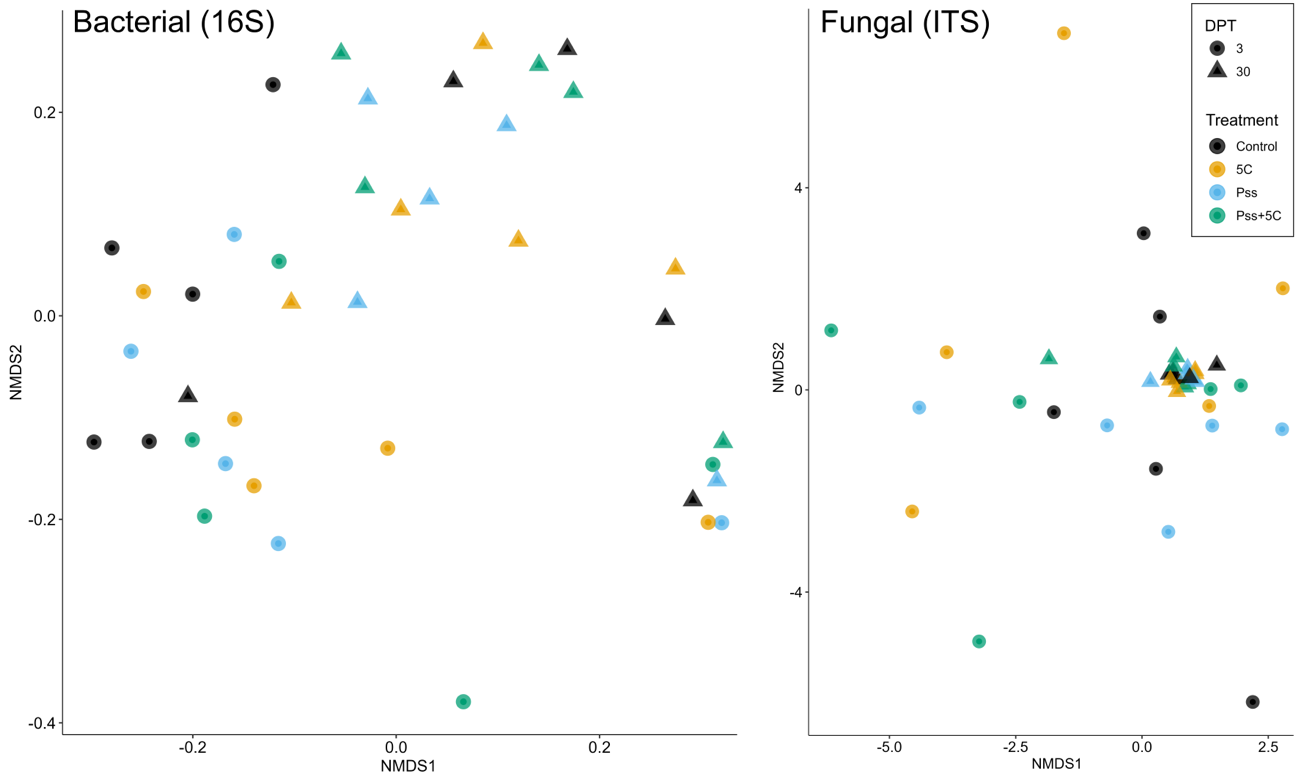


**Figure S12.** **The first two dimensions of non-metric multidimensional scaling of bacterial and fungal Bray-Curtis beta diversity indices in relation to the treatment and sampling time 3 and 30 day post treatment (DPT).**

**
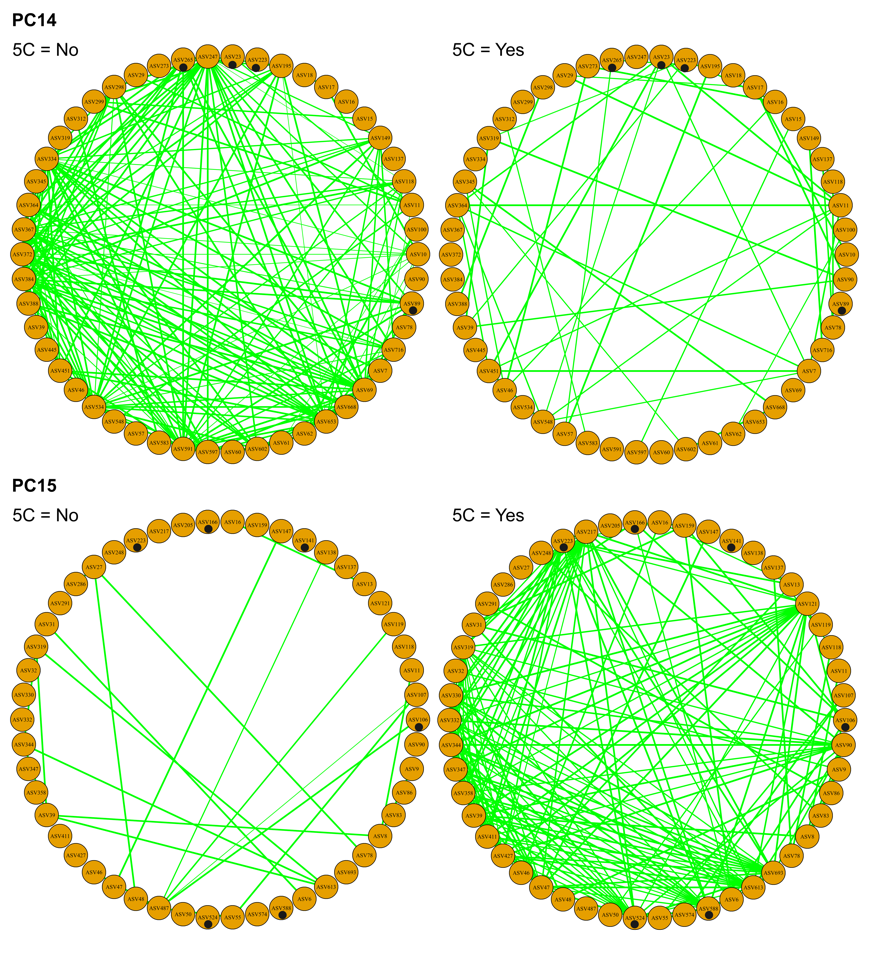
**

**Figure S13. Co-occurrence network of selected bacterial (16S) ASVs in samples that received 5C treatment (5C=Yes) or not (5C=No).**The 50 ASVs with the highest absolute loadings of principal component (PC) 14 and 15 are shown as nodes. The edges represent significant (p<0.005) positive (green) or negative (red) Pearson correlation. The thickness of the edges corresponds to the strength of the correlation. Only correlations with a coefficient >0.3 are shown. ASVs belonging to *Pseudomonas* genus, with above 50% confidence are denoted with a black dot. In total PC14 and PC15 represented 2 % and 1.8 % of the total variance. Application of 5C affected PC14 independently of sampling time and *Pss* co-application (significant 5C main effect, df=1, F=8.82, p=0.006). The application of 5C reduced the number of positive correlations of two potential *Pseudomonas syringae* ASV265 and ASV89 (99.7% similarity with *P*. *syringae*) and increased the number of positive correlations between a ‘non - *syringae’* *Pseudomonas* ASV23 and other top 50 ASVs in PC14. 5C affected PC15 as a three-way interaction of 5C with *Pss* and sampling which means that the composition of communities represented by PC15 changed differently in different *Pss* x sampling time combinations. Co-occurrence of PC15 showed more positive correlations between ASVs in samples that received 5C. Among the top 50 ASVs that increased number of positive correlations were 5 *Pseudomonas* genus ASVs (ASV106, ASV166, ASV223, ASV524 and ASV588) which all seem to be non-*P*. *syringae* based on Blastn analysis. They all shared below 98% identity with *P. syringae* strains and above 99.5% identify with non-*P. syringae* strains.


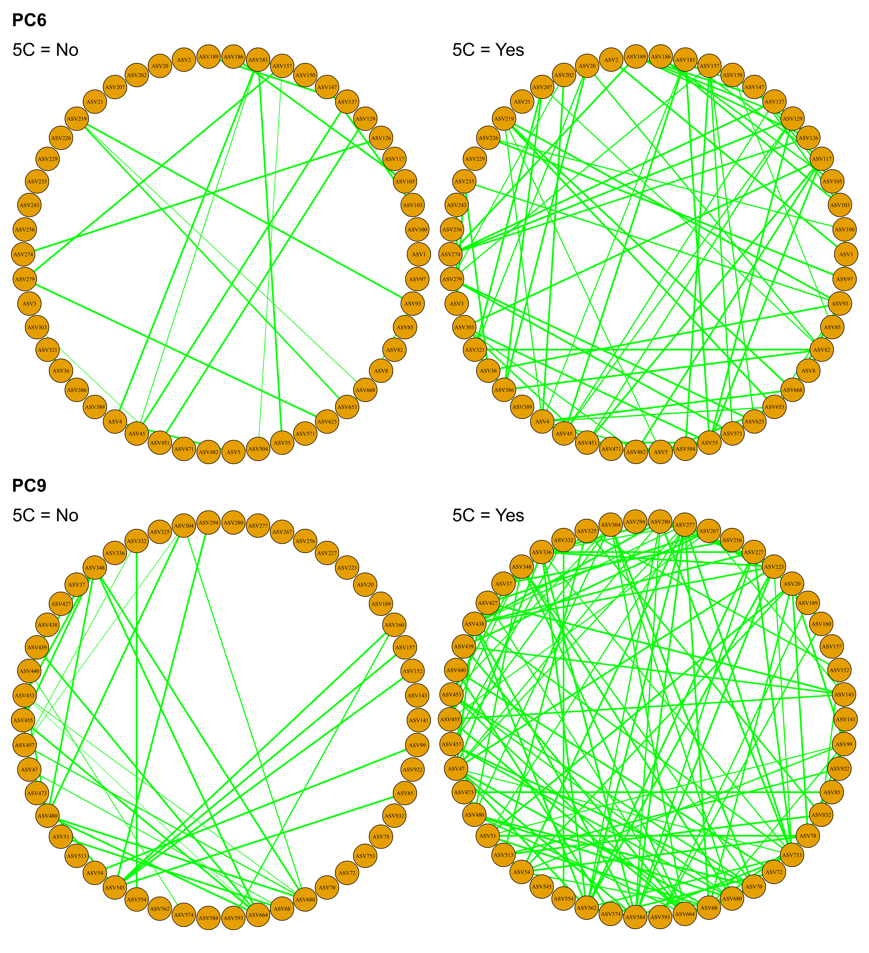


**Figure S14. Co-occurrence network of selected fungal (ITS) ASVs in samples that received 5C treatment (5C=Yes) or not (5C=No).**The 50 ASVs with the highest absolute loadings of principal component (PC) 6 and 9 are shown as nodes. The edges represent significant (p<0.005) positive (green) or negative (red) Pearson correlation. The thickness of the edges corresponds to the strength of the correlation. Only correlations with coefficient >0.3 are shown. Application of 5C had a small but significant effect on fungal PC6 (2.9% total variation) in a *Pss*-dependant manner (significant *Pss* x 5C interaction, df=1, F=4.68, p= 0.04) and on PC9 (2.6% total variation) where the effect of 5C was dependant on a combination of *Pss* application and sampling time (significant time x *Pss* x 5C interaction, df=1, F=10.03, p=0.005). In both cases we observed higher frequency of positive correlations in ASV networks constructed with samples that received 5C compared to the samples that did not receive 5C.

**Table S1. Statistical analysis of figure 2, 3, 6 and 7 in the main part of the results.** To evaluate differences in the killing curves and growth curves of the bacterial isolates in the absence and presence of phages, individually or in cocktail 5C, ANOVA test was used at specific time points. Post hoc Tukey test were applied to evaluate differences among treatments (p<0.05). All the statistical analysis were carried out in GraphPad prism 9 (Boston, Massachusetts USA, [www.graphpad.com](http://www.graphpad.com)). The data are supplied in a separate Excel spreadsheet.

| **Table S2. Summary of sequencing reads per sample or ASV and total bacterial (16S) and fungal (ITS) ASVs found in 40 leaf samples from 3 and 30 days post treatments.** | | | | |
| --- | --- | --- | --- | --- |
|  | **16S** | | **ITS** | |
|  | **raw** | **normalised** | **raw** | **normalised** |
| Total reads | 1329244 | 1126394 | 2357432 | 4021595 |
| Mean reads/sample | 33231.1 | 28159 | 58935.8 | 100539.9 |
| Median reads/Sample | 35406 | 16581 | 60970 | 89469.68 |
| Min reads/sample | 1784 | 3557 | 15544 | 622.0689 |
| Max reads/Sample | 67563 | 127467 | 75383 | 355253.2 |
| Mean reads/ASV | 2352 | 1993 | 3372 | 5753 |
| Median reads/ASV | 187 | 102 | 242 | 217 |
|  |  |  |  |  |
| Total ASVs | 565 | | 699 | |

| **Table S3: Top 10 bacterial (16s) and fungal (ITS) ASVs in 40 leaf samples from 3 and 30 day post treatments;** k: kingdom, o: order, f: family, g: genus. | | | | |
| --- | --- | --- | --- | --- |
| Amplicon | ASV no. | Counts | Proportion of total reads | Taxonomy (above 80% confidence) |
| 16s | ASV2 | 518180.7 | 46.0% | *Polaromonas* (g) |
|  | ASV1 | 385327 | 34.2% | *Phyllobacteriaceae* (f) |
|  | ASV6 | 11099.51 | 1.0% | *Hydrobacter* (g) |
|  | ASV5 | 8457.065 | 0.8% | *Bradyrhizobium* (g) |
|  | ASV14 | 7470.091 | 0.7% | *Azospirillum* (g) |
|  | ASV45 | 5356.733 | 0.5% | *Taibaiella* (g) |
|  | ASV166 | 4985.971 | 0.4% | *Pseudomonas* (g) |
|  | ASV53 | 4632.201 | 0.4% | *Achromobacter* (g) |
|  | ASV30 | 4358.557 | 0.4% | *Paracoccus* (g) |
|  | ASV137 | 4264.612 | 0.4% | *Bacteria* (k) |
| ITS | OTU1 | 1789827 | 44.5% | *Taphrina* |
|  | OTU3 | 283439.1 | 7.0% | *Taphrina* |
|  | OTU2 | 213622.9 | 5.3% | *Dothideales* (o) |
|  | OTU4 | 197437.6 | 4.9% | *Filobasidium* |
|  | OTU5 | 162281.2 | 4.0% | *Phaeotremellaceae* (f) |
|  | OTU6 | 143330.1 | 3.6% | *Vishniacozyma* |
|  | OTU8 | 116624.8 | 2.9% | *Exophiala* (g) |
|  | OTU7 | 80337.07 | 2.0% | *Monilinia* (g) |
|  | OTU10 | 67951.01 | 1.7% | *Cladosporium* |
|  | OTU13 | 63303.47 | 1.6% | *Sporidiobolus* |

## Table S4. Sequences of *Pseudomonas* ASVs with significantly different abundance across time and treatments.

| >ASV28 |
| --- |
| TGGGGAATATTGGACAATGGGCGAAAGCCTGATCCAGCCATGCCGCGTGTGTGAAGAAGGTCTTCGGATTGTAAAGCACTTTAAGTTGGGAGGAAGGGCAGTTACCTAATACGTGATTGTTTTGACGTTACCGACAGAATAAGCACCGGCTAACTCTGTGCCAGCAGCCGCGGTAATACAGAGGGTGCAAGCGTTAATCGGAATTACTGGGCGTAAAGCGCGCGTAGGTGGTTTGTTAAGTTGAATGTGAAATCCCCGGGCTCAACCTGGGAACTGCATCCAAAACTGGCAAGCTAGAGTATGGTAGAGGGTGGTGGAATTTCCTGTGTAGCGGTGAAATGCGTAGATATAGGAAGGAACACCAGTGGCGAAGGCGACCACCTGGACTGATACTGACACTGAGGTGCGAAAGCGTGGGGAGCAAACAGG |
| >ASV166 |
| TGGGGAATATTGGACAATGGGCGAAAGCCTGATCCAGCCATGCCGCGTGTGTGAAGAAGGTCTTCGGATTGTAAAGCACTTTAAGTTGGGAGGAAGGGCAGTAAATTAATACTTTGCTGTTTTGACGTTACCGACAGAATAAGCACCGGCTAACTCTGTGCCAGCAGCCGCGGTAATACAGAGGGTGCAAGCGTTAATCGGAATTACTGGGCGTAAAGCGCGCGTAGGTGGTTCGTTAAGTTGGATGTGAAATCCCCGGGCTCAACCTGGGAACTGCATTCAAAACTGACGAGCTAGAGTATGGTAGAGGGTGGTGGAATTTCCTGTGTAGCGGTGAAATGCGTAGATATAGGAAGGAACACCAGTGGCGAAGGCGACCACCTGGACTGATACTGACACTGAGGTGCGAAAGCGTGGGGAGCAAACAGG |
| >ASV23 |
| TGGGGAATATTGGACAATGGGCGAAAGCCTGATCCAGCCATGCCGCGTGTGTGAAGAAGGTCTTCGGATTGTAAAGCACTTTAAGTTGGGAGGAAGGGCAGTAAGCGAATACCTTGCTGTTTTGACGTTACCGACAGAATAAGCACCGGCTAACTCTGTGCCAGCAGCCGCGGTAATACAGAGGGTGCAAGCGTTAATCGGAATTACTGGGCGTAAAGCGCGCGTAGGTGGTTTGTTAAGTTGAATGTGAAATCCCCGGGCTCAACCTGGGAACTGCATCCAAAACTGGCAAGCTAGAGTAGGGCAGAGGGTGGTGGAATTTCCTGTGTAGCGGTGAAATGCGTAGATATAGGAAGGAACACCAGTGGCGAAGGCGACCACCTGGGCTCATACTGACACTGAGGTGCGAAAGCGTGGGGAGCAAACAGG |
